# An integrative bioinformatics analysis for identifying hub genes associated with infection of lung samples in patients infected with SARS-CoV-2

**DOI:** 10.1186/s40001-021-00609-4

**Published:** 2021-12-17

**Authors:** Tian-Ao Xie, Zhi-Jian He, Chuan Liang, Hao-Neng Dong, Jie Zhou, Shu-Jin Fan, Xu-Guang Guo

**Affiliations:** 1 Department of Clinical Laboratory Medicine, The Third Affiliated Hospital of Guangzhou Medical University, Guangzhou, 510150 China; 2 Department of Clinical Medicine, The Third Clinical School of Guangzhou Medical University, Guangzhou, 511436 China; 3 Key Laboratory for Major Obstetric Diseases of Guangdong Province, The Third Affiliated Hospital of Guangzhou Medical University, Guangzhou, 510150 China; 4 Key Laboratory of Reproduction and Genetics of Guangdong Higher Education Institutes, The Third Affiliated Hospital of Guangzhou Medical University, Guangzhou, 510150 China

**Keywords:** SARS-CoV-2, Hub genes, Protein–protein interactions network, Differentially expressed genes

## Abstract

**Background:**

At the end of 2019, the world witnessed the emergence and ravages of a viral infection induced by severe acute respiratory syndrome coronavirus 2 (SARS-CoV-2). Also known as the coronavirus disease 2019 (COVID-19), it has been identified as a public health emergency of international concern (PHEIC) by the World Health Organization (WHO) because of its severity.

**Methods:**

The gene data of 51 samples were extracted from the GSE150316 and GSE147507 data set and then processed by means of the programming language R, through which the differentially expressed genes (DEGs) that meet the standards were screened. The Gene Ontology (GO) and Kyoto Encyclopedia of Genes and Genomes (KEGG) analyses were performed on the selected DEGs to understand the functions and approaches of DEGs. The online tool STRING was employed to construct a protein–protein interaction (PPI) network of DEGs and, in turn, to identify hub genes.

**Results:**

A total of 52 intersection genes were obtained through DEG identification. Through the GO analysis, we realized that the biological processes (BPs) that have the deepest impact on the human body after SARS-CoV-2 infection are various immune responses. By using STRING to construct a PPI network, 10 hub genes were identified, including IFIH1, DDX58, ISG15, EGR1, OASL, SAMD9, SAMD9L, XAF1, IFITM1, and TNFSF10.

**Conclusion:**

The results of this study will hopefully provide guidance for future studies on the pathophysiological mechanism of SARS-CoV-2 infection.

**Supplementary Information:**

The online version contains supplementary material available at 10.1186/s40001-021-00609-4.

## Introduction

Currently, a new type of coronavirus, first named as Coronavirus 2019 (COVID-19), has spread rapidly in 212 countries. As of May 25, 2020, more than 5.5 million cases have been diagnosed and over 340,000 people have died of it. The results of genome sequencing have unveiled that this pneumonia is induced by a new type of coronavirus, namely severe acute respiratory syndrome coronavirus 2 (SARS-CoV-2) [1].

At the beginning of the outbreak, scientists believed that the disease was first spread from animals to humans, and then from symptomatic people to other humans until the first human-to-human transmission from asymptomatic carriers recorded in Germany [2–4]. It has been proved that the new CoV can spread from person to person through breathing droplets. It is worth noting that the respiratory tract may not be the only route of transmission. Specifically, the direct or even indirect contact with the mucous membranes of eyes, mouth, or nose can also spread SARS-CoV-2 [5, 6]. Besides, as documented by a research lately, it is possible that SARS-CoV-2 infection can be spread through the digestive tract [7].

As people in all age groups are vulnerable to SARS-CoV-2, those over the middle age prove to be the most susceptible. A majority of patients admitted to hospital for the diagnosis of COVID-19 average from 48 to 58 years [8]. After SARS-CoV-2 infects human body, it enters the alveolar epithelial cells to rapidly replicate and trigger a powerful immunological reaction, causing damage to lung tissues and the cytokine storm syndrome, which is an important cause of acute respiratory distress syndrome (ARDS) and multiple organ failure [9, 10]. At present, in clinical practice, the infection prevention and control methods are implemented, as well as the supportive nursing is provided for patients. However, no vaccine or specific treatment has been successfully developed for SARS-CoV-2 [11].

Since the emergence of COVID-19, it has been extensively documented by scholars and researchers from all over the world. Recent studies have shown that spike protein is of vital importance in inducing neutralizing antibodies. Vaccines can be developed to specifically identify the spike proteins of SARS and angiotensin-converting enzyme 2 (ACE2) receptors [12]. Although the SARS-CoV-2 and SARS spike protein sequences show some overlaps, viral genetic mutations and increased antibody dependence may affect the efficacy of the vaccine [13]. In order to reduce the occurrence of above situation, it is a good approach to screen out hub genes that are closely related to the pathological process of SARS-CoV-2 invasion into lung cells, and to understand the changes in host cell molecular level during this interaction.

With the technology of microarray and high-throughput sequencing evolving increasingly, genes related to the occurrence, development, diagnosis and treatment of diseases can be identified. In order to better understand the important genes of alveolar epithelial cells that SARS-CoV-2 acts on and to provide more information for vaccine development, this study employed integrated bioinformatics methods to conduct cross-platform research and large-sample survey [14].

## Methods and materials

### Study process

In this study, we mainly carried out DEGs screening, GO analysis, KEGG analysis, PPI network establishment and statistical verification for gene set data. The specific process is shown in Additional file 1: Figure S1.

### Data sources

The Gene Expression Omnibus (GEO) is an open database that stores expression chip data, from which the GSE150316 and the GSE147507 gene expression profiles were obtained. The GSE150316 gene expression profile was obtained from the platform GPL18573 Illumina NextSeq 500 (Homo sapiens), and the GSE147507 gene expression profile was obtained from the analysis of platform GPL18573 Illumina NextSeq 500 (Homo sapiens) and platform GPL28369 Illumina NextSeq 500 (*Mustela putorius furo*). From the GSE150316 and GSE147507 gene expression profiles, the gene data of 47 samples and that of four samples were selected, respectively, both of which were included for research and analysis. Out of the gene data of 47 samples, 42 were obtained from the lung tissues of 11 COVID-19-positive patients, and five from normal human lung tissues. Out of the gene data of four samples, two were from lung tissue samples of the same COVID-19 positive patient (technical replication), and two were from lung tissues of two normal individuals (a male and a female).

### Data processing

The system matrix files and other related files of GSE150316 and GSE147507 were downloaded from the GEO database. R was deployed to convert the counts of these genes into TPM files, and the limma package was used in R language to standardize the data of each group and select the DEGs that meet the standards [15]. The GSE150316 data set is based on |log_2_ FC|≥ 1, p-value < 0.05 as the standard, and the standard of the GSE147507 data set is |log2 FC|≥ 2, p-value < 0.01—both meet the statistical standard. Therefore, this standard was adopted to distinguish genes with significant changes in expression fold change from other genes. All eligible DEGs were included in this study [16].

### Identification of DEGs

The GO analysis and the KEGG enrichment analysis, which can perform functional enrichment analysis and pathway enrichment analysis on DEGs, respectively, were employed to delve into the biological functions of DEGs. GO is a biological information resource that stores computable knowledge of the functions of genes and gene products and describes the biological functions of genes and gene products in living bodies through annotations that have been confirmed by relevant studies, and it has become the main annotation method for high-throughput sequences [17, 18]. In the GO analysis, it is widely accepted that p < 0.05 is statistically significant. This step enables the identification of genes that have significant effects in the biological process (BP), cellular component (CC), and molecular function (MF) [19]. KEGG, which is composed of the PATHWAY database, LIGAND database, and GENES database, is a resource library with genomic sequences and related molecular data obtained from other high-throughput experimental analyses. KEGG boasts of powerful image tools that clearly display various biological metabolic pathways and the connections between them. In the KEGG analysis, p < 0.05 is considered to be of statistical significance [20].

### Construction of the protein–protein interaction (PPI) network and identification of hub genes

The PPI network consists of individual proteins that interact with each other to be involved in a variety of life activities, such as the transmission of biological signals, regulation of gene expression, metabolism of energy and substance, as well as the regulation of cell cycle. Analyzing protein interaction networks allows us to better understand how proteins work and function in biological systems. Therefore, the online tool STRING (version 11.0) (http://string-db.org/) (medium confidence > 0.4), to which the DEGs were uploaded, was employed to construct the PPI network. Following this, the PPI network was visualized with the help of the software Cytoscape (version 7.3.2) (the default parameters). Hub genes were defined as those with a degree in the top 10.

### Performing non-paired t-test for verification

To ensure that the 10 hub genes obtained in this study are worthy of further study, the data of GSE150728 were used to verify them. The data of the control group and the infection group of these 10 hub genes in GSE150728 were extracted and a non-paired t-test analysis was performed using GraphPad Prism (version 8.0.2). p-value < 0.05 was considered to be statistically significant.

## Result

### The DEGs among GSE150316 and GSE147507

**Fig. 1:**
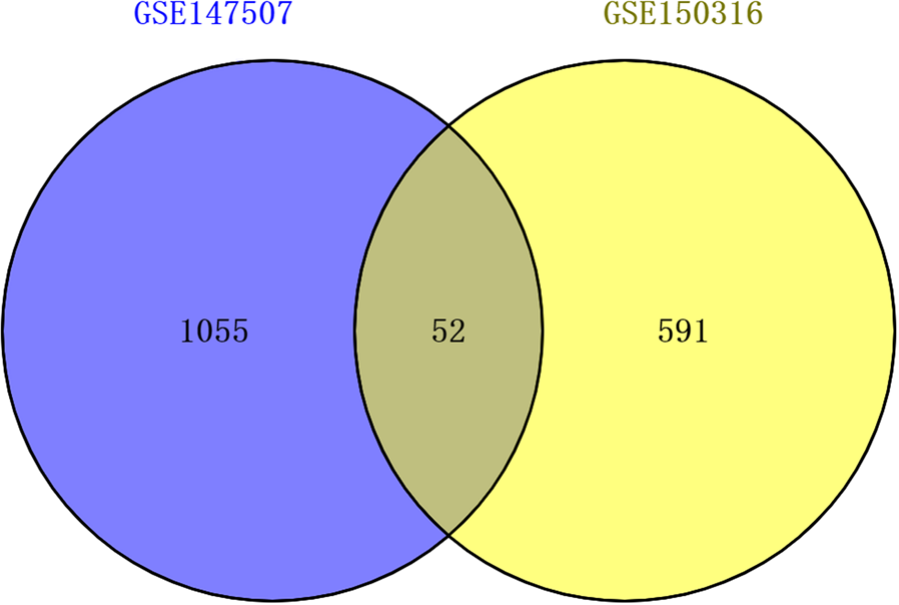
The intersection DEGs of GSE147507 and GSE150316

**Fig. 2:**
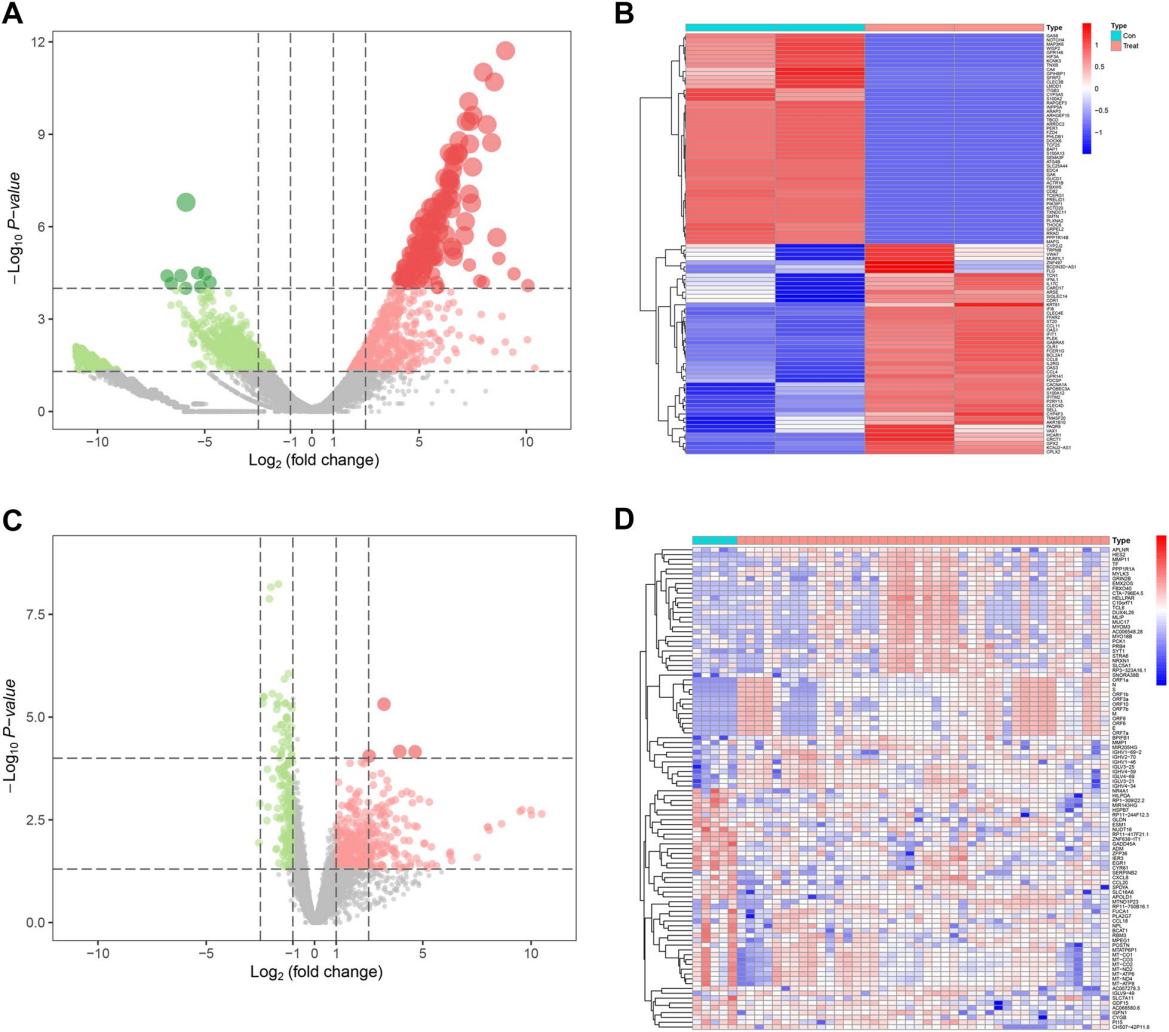
Volcano map and heat map of DEGs. **A**, **B** The volcano map and heat map of DEGs that were plotted for GSE147507. **C** **D** The volcano map and heat map of DEGs that were plotted for GSE150316. The *X axis* represents the logarithm of the fold change. The *Y axis* represents the negative value of the logarithm of the *p* value. Red dots represent up-regulated genes that meet the screening criteria, and blue dots represent down-regulated genes that meet the screening criteria (**A**, **C**). Gene expression data are converted into a data matrix. Each column represents the genetic data of a sample, and each row represents a gene. The color of each cell represents the expression level, and there are references to expression levels in different colors in the upper right corner of the figure (**B**, **D**)

The gene data of 51 samples were obtained from GSE150316 and GSE147507 gene expression profiles. Detailed information about the data sources of this research is described in Table 1. First, according to abs |log FC|> 2 and p-value < 0.01, 1107, DEGs were selected from the gene data of four samples of GSE147507. Among them, 384 genes were up-regulated and 723 genes were down-regulated. According to abs |log FC|> 1, p-value < 0.05, 643, DEGs were selected from the gene data of 47 samples of GSE150316. Among them, 449 genes were up-regulated and 194 genes were down-regulated. Additionally, there are 52 intersection genes between GSE147507 and GSE150316 (refer to Additional file 2: Table S1 and Fig. 1). Details of their function and fold change are presented in Additional file 3: Table S2 (The above information of gene functions comes from GeneCards) (http://www.genecards.org). Then, the volcano map and the heat map of the DEGs were plotted for GSE147507 and GSE150316 (refer to Fig. 2A-D).

**Table 1:** Details of the data sources for this study

Gene expression profile	Sample collection	Sample genetic data included	Platform
GSE147507	Lung tissue	GSM4462413-16	GPL18573 Illumina NextSeq 500 (Homo sapiens)
GSE150316	Lung tissue	GSM4546576-79 GSM4546581-82 GSM4546584 GSM4546586 GSM4546588-89 GSM4546592 GSM4546596-99 GSM4546601 GSM4546608-12 GSM4698531-40 GSM4698544-53 GSM4698521-23 GSM4698526-28	GPL18573 Illumina NextSeq 500 (Homo sapiens)

### Enrichment analysis of the pathway and process in SARS-CoV-2 infection

**Fig. 3:**
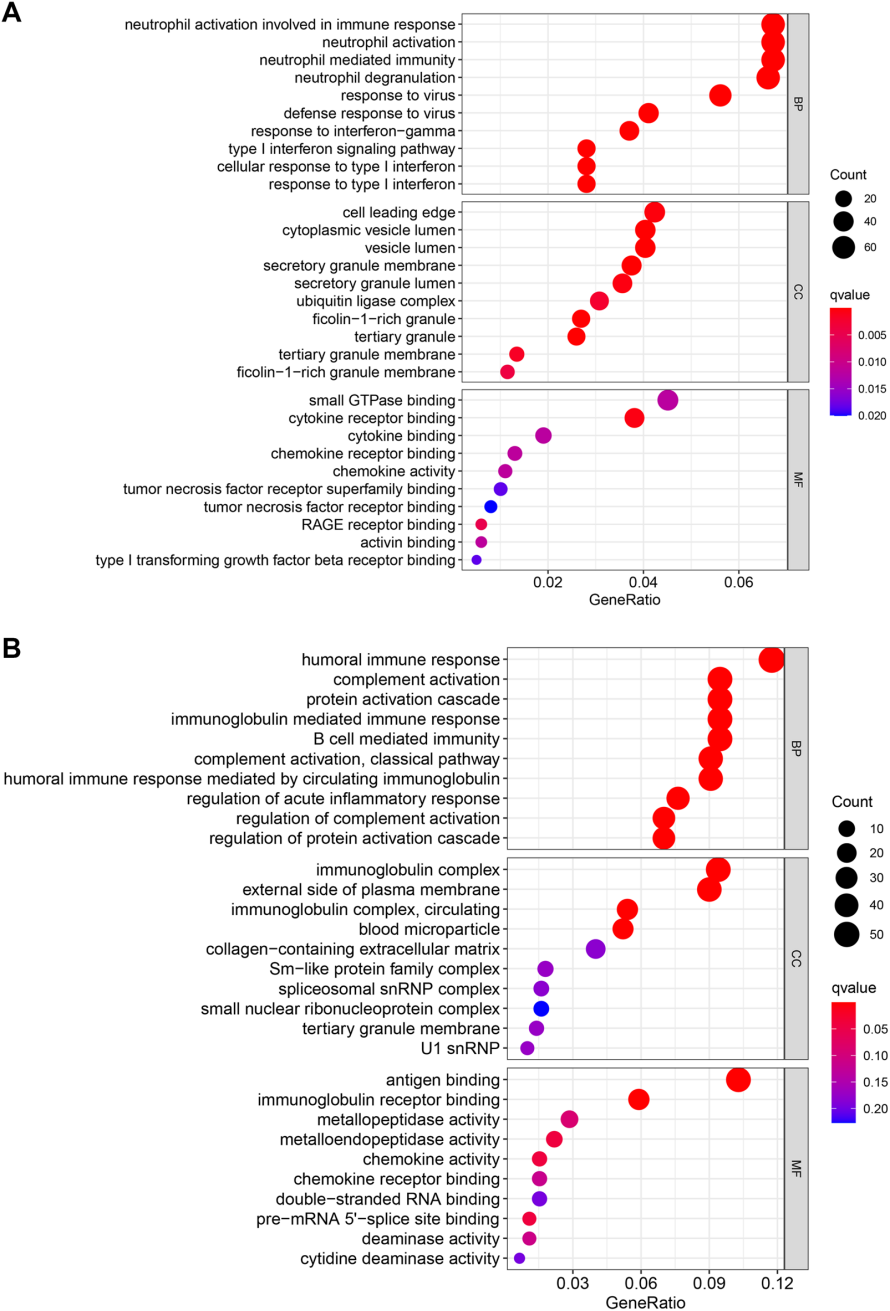
The top 10 enriched terms of GO analysis (BP, CC, MF) in the system matrix file GSE147507 (**A**) and GSE150316 (**B**)

The GO analysis demonstrated that in GSE147507, the parts that exert significant influence on the annotation of biological processes (BP) are the neutrophil activation involved in immune response and neutrophil activation. Moreover, in GSE150316, humoral immune response and complement activation have significant effects on the annotation of biological processes (BP). Those parts that are statistically significant in the three processes of GO analysis, BP, CC, and MF, are shown in Fig. 3A, B, respectively. Additional file 3: Table S2 shows a collection of the 20 most important GO enrichment items in the BP of GSE147507 and GSE150316.

### Analysis of the KEGG pathway

**Fig. 4:**
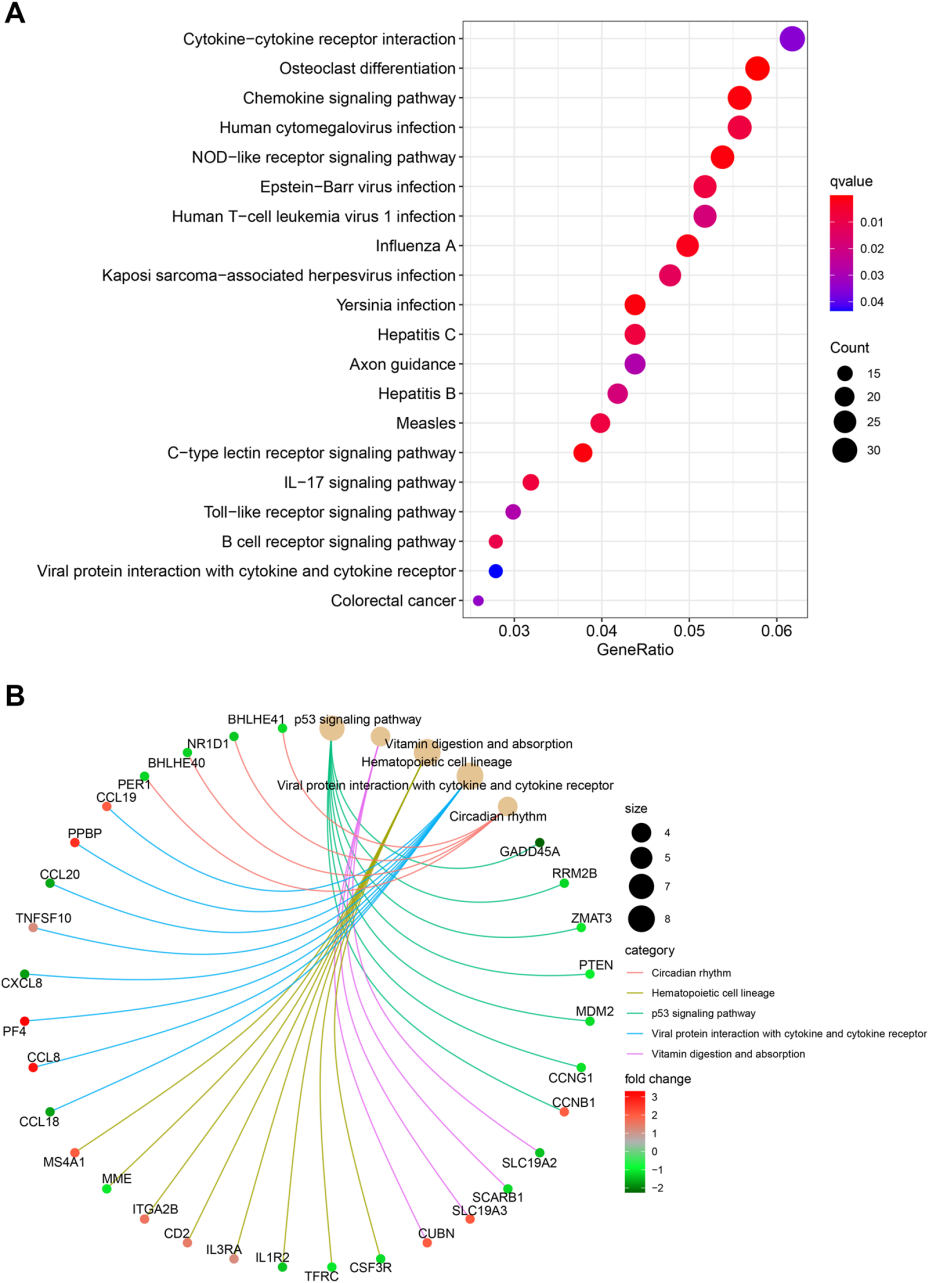
Functional and pathway enrichment analyses of DEGs in the system matrix file GSE147507 and GSE150316

In order to better identify the biological function of DEGs, an in-depth analysis of DEG was conducted by means of KEGG, where p-value < 0.05 was considered to be statistically significant. In system matrix files GSE147507, the results demonstrated that 20 meaningful approaches were analyzed in total. The cell signaling pathways significantly associated with the SARS-CoV-2 infection include, but are not limited to osteoclast differentiation, chemokine signaling pathway, Yersinia infection, NOD-like receptor signaling pathway, and C-type lectin receptor signaling pathway. The most abundant KEGG path information of the system matrix files GSE147507 and GSE150316 is shown in Additional file 4: Table S3. However, the KEGG path information of GSE150316 is not obvious, and it is not shown in Additional file 4: Table S3. All the specific enrichment pathways obtained from the analysis of the DEGs of the system matrix file GSE147507 are shown in Fig. 4A. In the system matrix file GSE150316, we did not obtain highly significant signal pathway results after analysis, but, as can be seen in Fig. 4B, it may be related to the circadian rhythm, hematopoietic cell lineage, p53 signaling pathway, viral protein interaction with cytokine and cytokine receptor, and vitamin digestion and absorption.

### Hub genes identification with DEGs protein–protein interaction network (PPI)

**Fig. 5:**
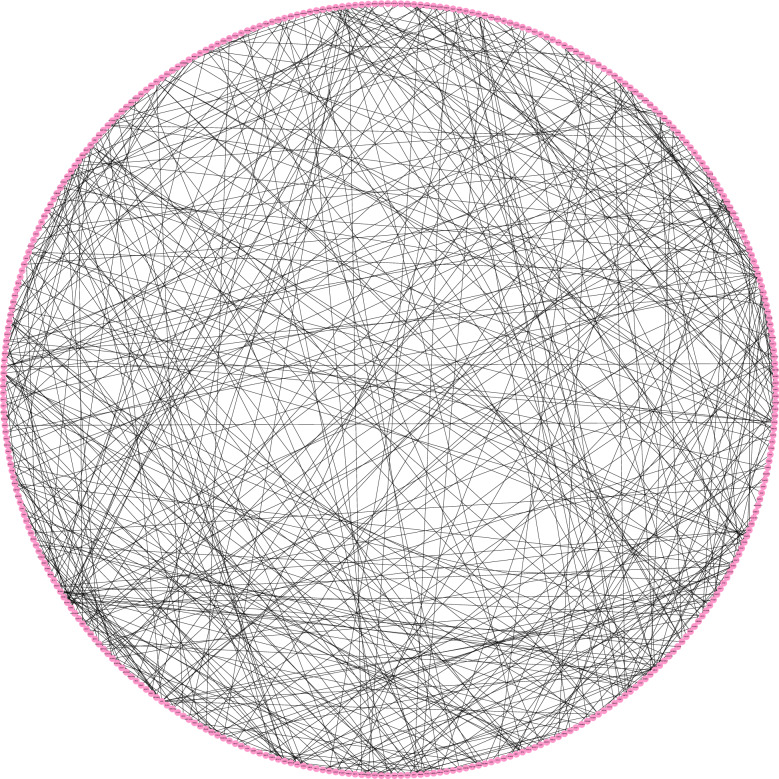
The PPI network of GSE147507

**Fig. 6:**
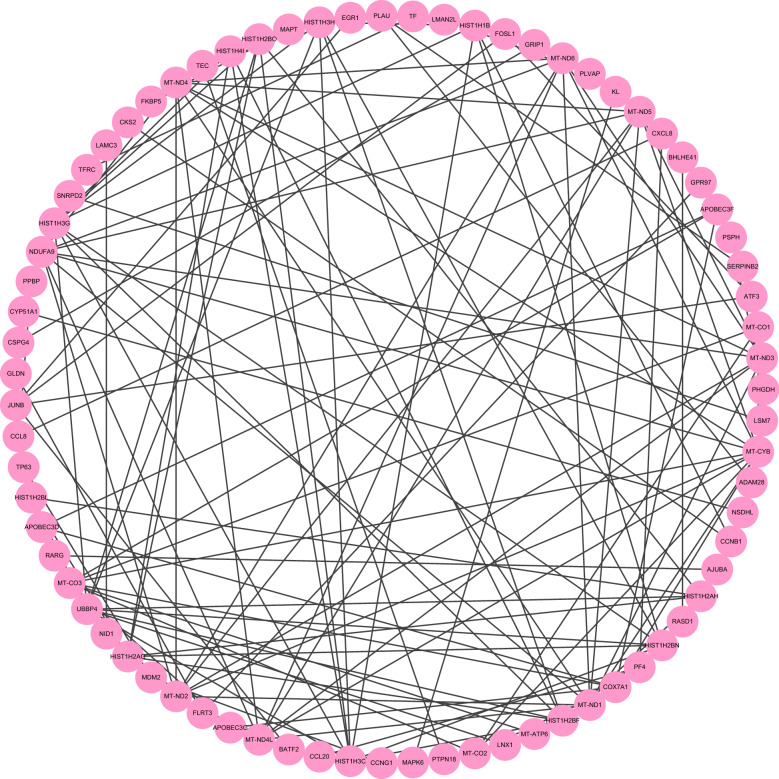
The PPI network of GSE150316

**Fig. 7:**
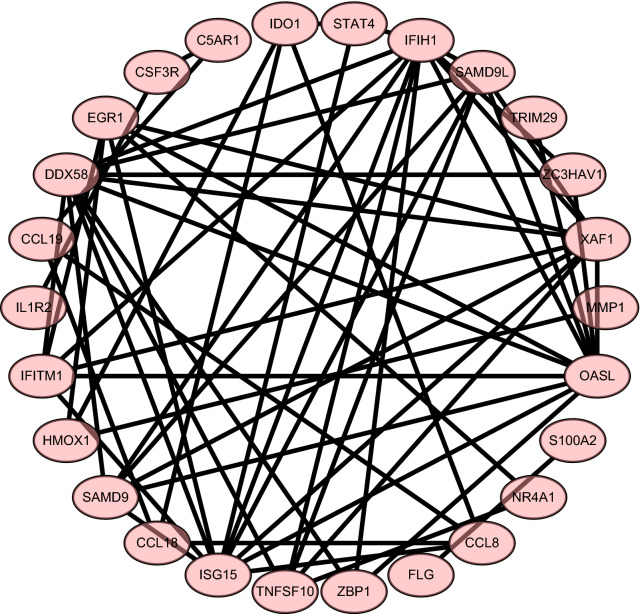
The intersection PPI network of GSE147507 and GSE150316

**Fig. 8:**
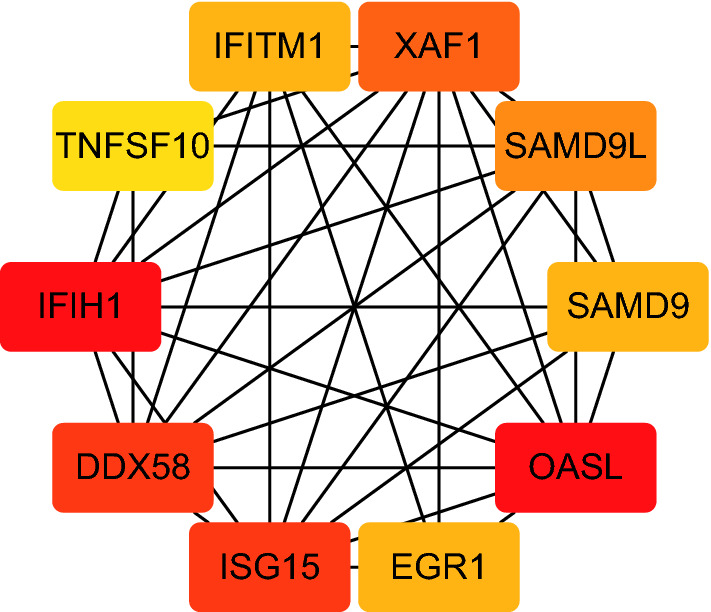
The hub genes of GSE147507 and GSE150316

From a physiological point of view, proteins rarely function on their own and usually interact with each other within a network. Therefore, this research constructed the PPI network on STRING (version 11.0). Finally, it was observed that the PPI network of GSE147507 has 371 nodes and 442 edges (refer to Fig. 5), the PPI network of GSE150316 has 75 nodes and 122 edges (refer to Fig. 6), and the PPI network of intersection has 26 nodes and 59 edges (Fig. 7). In addition, the PPI network was visualized using Cytoscape (version 7.3.2), and the 10 hub genes were identified as IFIH1, DDX58, ISG15, EGR1, OASL, SAMD9, SAMD9L, XAF1, IFITM1, and TNFSF10 (Fig. 8). Their functions are shown in Table 2.

**Table 2:** The function of 10 hub genes

ID	Function
IFIH1	Encoding MDA5 which is an intracellular sensor of viral RNA that triggers the innate immune response, involves in a proinflammatory response that includes interferons and plays an important role in enhancing natural killer cell function in malaria infection
DDX58	Involving in viral double-stranded (ds) RNA recognition and the regulation of the antiviral innate immune response
ISG15	Including chemotactic activity towards neutrophils, direction of ligated target proteins to intermediate filaments, cell-to-cell signaling, and antiviral activity during viral infections
EGR1	Encoding a nuclear protein which functions as a transcriptional regulator
OASL	Diseases associated with OASL include West Nile Fever and West Nile Virus Infection
SAMD9	Encoding a sterile alpha motif domain-containing protein that may play a role in regulating cell proliferation and apoptosis
SAMD9L	Encoding a cytoplasmic protein that acts as a tumor suppressor but also plays a key role in cell proliferation and the innate immune response to viral infection
XAF1	Encoding a protein which binds to and counteracts the inhibitory effect of a member of the IAP (inhibitor of apoptosis) protein family. (IAP proteins bind to and inhibit caspases which are activated during apoptosis)
IFITM1	Involving Interferon gamma signaling and Immunoregulatory interactions between a Lymphoid and a non-Lymphoid cell
TNFSF10	Encoding a cytokine that preferentially induces apoptosis in transformed and tumor cells, but does not appear to kill normal cells

### Verification of the hub gene through non-paired t-test

**Fig. 9:**
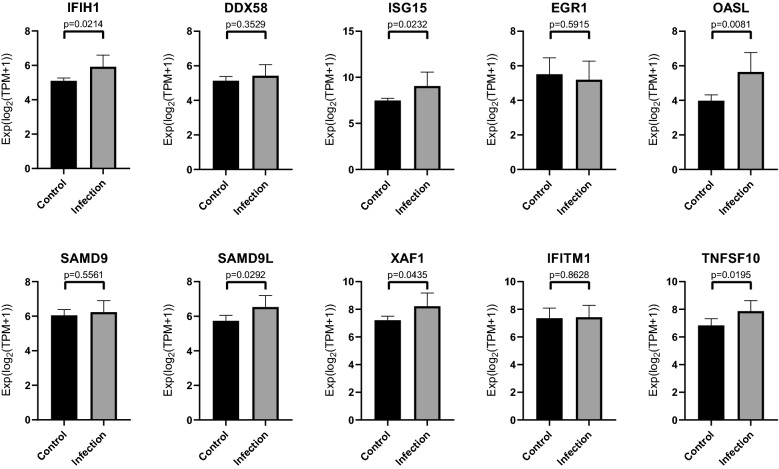
The verification of hub genes

The results of the t-test are demonstrated in Fig. 9. There are six hub genes that have statistical significance: IFIH1, SAMD9L, ISG15, XAF1, OASL, and TNFSF10. The p-values of SAMD9, DDX58, EGR1, and IFITM1 were 0.5561, 0.3529, 0.5915, and 0.8628, respectively, which are all greater than 0.05. Thus, they were not statistically significant. This study will not conduct further research on these four hub genes.

## Discussion

At the beginning of 2020, SARS-CoV-2 triggered a worldwide outbreak of COVID-19 pneumonia, rendering it imperative to acquire better knowledge about SARS-CoV-2 and create a vaccine to prevent its spread among the populace [21]. Therefore, uncovering the potential molecular mechanism of COVID-19 is of paramount importance. The gene expression profile of the GSE150316 and GSE147507 data sets were used to screen DEGs. As a result, 1107 DEGs were identified in the GSE147507, including 384 up-regulated DEGs and 723 down-regulated DEGs, and 643 DEGs were identified in the GSE150316, including 449 up-regulated DEGs and 194 down-regulated DEGs. The GO analysis was utilized to perform a functional enrichment analysis on the DEGs obtained, the results of which demonstrated the significantly enriched disease-related BP, CC, and MF. Meaningful enrichment was also reported through the KEGG analysis. Next, the PPI network construction, module analysis, and central gene identification were performed to screen a total of 10 important hub genes that may play a key regulatory role in the pathophysiology of COVID-19.

As shown in the graph describing the results of the GO analysis, it is generally believed that the smaller the *p*-value of the GO item, the more significant the enrichment of DEG in the GO item. For GSE147507, the functions of neutrophil activation involved in immune response, neutrophil activation and neutrophil-mediated immunity are the most significant parts of biological processes. The manner in which SARS-CoV-2 invades host cells is related to the angiotensin-converting enzyme 2 (ACE2), and SARS-CoV-2 invades human cells by binding to ACE2 [22]. The human body’s pattern recognition receptor recognizes the SARS-CoV-2 virus antigen and presents it to natural killer cells and CD8-positive cytotoxic T cells, which activate the body’s innate immunity and adaptive immunity and triggers a large number of pro-inflammatory cytokines and chemotaxis in the body factor generation. Some pro-inflammatory cytokines can activate neutrophils. With the development of COVID-19, the level of neutrophils in the blood continues to rise. Neutrophils participate in the immune response and fight against cells through the engulfment of microbes, the formation of reactive oxygen species, degranulation, the secretion of antimicrobials, and the formation of increased neutrophil extracellular traps [23, 24]. In terms of biological processes, the functions of the humoral immune response, complement activation, and protein activation cascade are the most significant for GSE150316. After the human body is infected with SARS-CoV-2, the humoral immune response and cellular immune response are activated. In the humoral immune response, plasma cells produce immunoglobulins that bind to antigens on the surface of SARS-CoV-2 to form an immunoglobulin complex. The immunoglobulin complex and the mannose-binding lectin that is involved in complement activation form the classical pathway and the lectin pathway, respectively. These two ways of activating complement are essentially involve cascade protein activation [25]. At the same time, immunoglobulin can also bind to the immunoglobulin receptor of the cells invaded by SARS-CoV-2 to perform phagocytosis and adhesion [22, 23, 26].

Based on the enrichment analysis of the KEGG pathway, it was found that certain cell signal transduction pathways are closely related to SARS-CoV-2 infection. When the human body is infected with the SARS-CoV-2 virus, the entry of SARS-CoV-2 into the alveolar epithelial cells causes the damage of lung tissue and leads to the uncontrolled production of pro-inflammatory cytokines [10, 11, 27]. SARS-CoV-2 mainly infects epithelial cells in the lung and results in the accumulation of white blood cells in injured or infected tissues. More importantly, the virus can enter macrophages and dendritic cells [28, 29]. The infection of these cells plays an important role in inducing pro-inflammatory cytokines that may cause disease [30]. In fact, many cytokines and chemokines are produced by macrophages and dendritic cells and their levels are elevated in the serum of patients infected by SARS-CoV-2 [31]. In response to injury or infection, the expression of the chemokine signaling pathways and the NOD-like receptor signaling pathways in the tissues is enhanced. In the signal transduction pathway of chemokines, the chemokines bind and activate chemokine receptors to embed the chemokine receptors in G protein-coupled receptors (GPCRs) in the cell membranes of leukocytes, thereby inducing leukocytes to change their adhesion and shape, adhere to the blood vessel wall, and penetrate the inflamed tissue along its chemotactic factor gradient [32]. The accumulated white blood cells remove pathogens and necrotic tissues through phagocytosis and proteolysis. In addition to their participation in leukocyte trafficking, chemokines can also produce a variety of other cells and participate in tissue responses, including proliferation, activation, differentiation, extracellular matrix remodeling, and angiogenesis, which may be related to tissue repair and reconstruction [33–36]. Moreover, some chemokines and receptors are constitutively expressed in specific tissues and cell types that can promote homeostasis, such as T cell development, stem cell migration, and lymphoid organogenesis [37]. When the SARS-CoV-2 virus invades human cells, it can also exert the body’s immune function through the processes of inflammasome assembly, signal transduction, transcription activation, and autophagy through the activated NOD-like receptor signal transduction pathway [38]. The NOD-like receptor signal transduction pathway and the Yersinia infection pathway can activate caspase-1 by the inflammasome of the multimeric protein complex. The activated caspase-1 leads to the processing and maturation of the pro-inflammatory cytokines, interleukin (IL)-1β and IL-18, which participate in the body’s immune response and lead to the death of specific inflammatory cells [39, 40]. Additionally, NOD1 and NOD2 can activate the serine/threonine kinase of NF-kB [41] and activate the mitogen-activated protein kinase (MAPK) signaling pathway, leading to the secretion of pro-inflammatory cytokines [42, 43]. It plays an important role in resisting SARS-CoV-2 infection and regulating host immune response. NOD2 can also sense the ssRNA of the virus and then activate it to produce interferon and antiviral defense.

The 10 pivotal genes screened through the PPI network were verified by the non-paired t-test, and four meaningless genes were found. The remaining six were all up-regulated genes that are closely related to the pathological process of SARS-CoV-2 infection in lung cells and may become new therapeutic targets or research directions. Over the past 15 years, ubiquitin-like protein ISG15 has been widely regarded as a major participant in the host antiviral response, and recent work has shown that it can directly inhibit viral replication and modulate host immunity [44]. OASL and SAMD9L have been shown to be important in viral infection and innate immunity, but the mechanism of their action is different from that of ISG15 [45, 46]. Studies have found that OASL makes the RNA detection system based on RIG-I more sensitive to viral RNA, which can be activated under viral infections that are relatively below the threshold level, and the SARS-Cov-2 virus in the body can be found faster [47]. The mechanism by which the expression of SAMD9L affects virus replication in varying degrees is not yet fully understood, but it has been reported that SAMD9L can inhibit West Nile virus replication [48, 49]. Similarly, the significant expression of IFIH1 and TSFSF10 is beneficial to the human body. Experiments have shown that the deficiency of IFIH1 can lead to primary immunodeficiency, which manifests as extreme susceptibility to common respiratory RNA viruses, such as human respiratory syncytial virus and rhinoviruses [50]. Moreover, the death receptor of TNFSF10 contributes to immune surveillance against viral infection by promoting apoptosis. It should be noted that we must pay close attention to determine whether SARS-CoV-2 escapes by regulating TNFSF10 receptor signal transduction [51]. Unlike the other five hub genes, the significant expression of XAF1 has a negative impact on the human body and may become a therapeutic target for COVID-19. From a previous study, we found that the expression of XAF1 was high in DENV2-infected VECs. After this expression of XAF1 was enhanced, as much as 28% of cells entered irreversible apoptosis 72 h after infection. Thus, it is very important to inhibit the expression of XAF1 during the treatment of COVID-19^52^.

There are certain limitations to this study. First, the conclusion is based on data collected from public databases, rather than from actual experiments. Enough clinical samples should be employed to ensure more accurate results. Second, the mechanism of several key genes in the pathological process of COVID-19 has not been fully understood. Therefore, further research and larger samples are needed.

## Conclusion

Our results indicate that the ten hub genes IFIH1, DDX58, ISG15, EGR1, OASL, SAMD9, SAMD9L, XAF1, IFITM1 and TNFSF10 may play an important regulatory role in SARS-CoV-2 infection. It may has guiding significance for researchers to study the infection mechanism of SARS-CoV-2 in the future.

## Supplementary Information

**Additional file 1: Figure S1.** Flowchart of data preparation, processing, and analysis in this study.

**Additional file 2: Table S1.** Up-regulated genes and down-regulated genes that meet the screening criteria.

**Additional file 3: Table S2.** The function and fold change of 52 identified genes.

**Additional file 4: Table S3.** BP pathways of GSE147507 and GSE150316 that are heavily enriched in GO analysis (ranked in the top 10 according to *p* value).

**Additional file 5: Table S4.** Ten most significantly enriched KEGG pathways in the system matrix file GSE147507 and GSE150316.

## Data Availability

Not applicable.
