# Supplementary figures and images for: An integrative bioinformatics analysis for identifying hub genes associated with infection of lung samples in patients infected with SARS-CoV-2
Source: Eur J Med Res. 2021 Dec 17;26:146. doi: 10.1186/s40001-021-00609-4 (PMC8677925; doi:10.1186/s40001-021-00609-4)

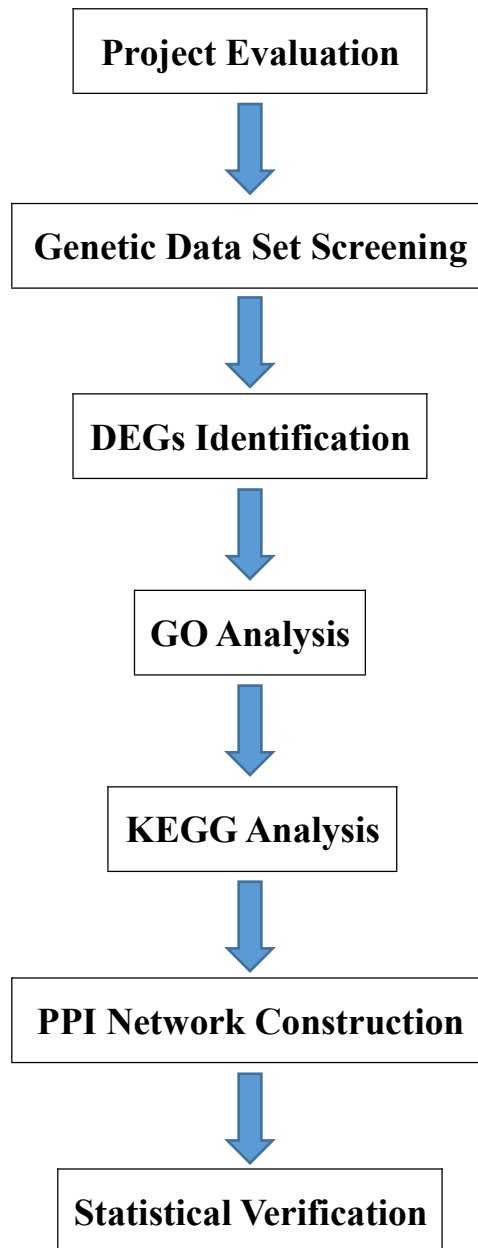

**Supplementary Figure S1. Flow chart of data preparation, processing, and analysis in this study.**

Supplement: Supplementary file 1 — Additional file 1: Figure S1. Flowchart of data preparation, processing, and analysis in this study. [file 40001_2021_609_MOESM1_ESM.pdf]
