# Supplementary material for: An integrative bioinformatics analysis for identifying hub genes associated with infection of lung samples in patients infected with SARS-CoV-2
Source: Eur J Med Res. 2021 Dec 17;26:146. doi: 10.1186/s40001-021-00609-4 (PMC8677925; doi:10.1186/s40001-021-00609-4)
Supplement: Supplementary file 2 — Additional file 2: Table S1. Up-regulated genes and down-regulated genes that meet the screening criteria. [file 40001_2021_609_MOESM2_ESM.docx]

**Supplementary Table S1. Up-regulated genes and down-regulated genes that meet the screening criteria.**

| Data set | DEGs | Gene terms | |
| --- | --- | --- | --- |
| GSE147507 | Up-regulated | LITAF/HLA-A/FKBP1A/STAT2/ANP32A/PLP2/C1QB/WIPF1/IFI30/APOL6/PFN1/CD55/PSMA4/EIF1B/SAT1/PSME3/PIGR/LY6E/CD164/SF3B4/CFL1/NME1/LMO2/SLC39A8/GPSM3/CAPZA1/CD4/RAB8B/NOP10/CD74/DCUN1D3/SPTLC2/PIM2/FHL3/C4orf3/IRF7/LPAR6/CRYAB/PPDPF/DYNLT1/CCRL2/USB1/CEACAM6/SMAP2/PDE4B/SERF2/PARP9/JAK3/HCK/NMI/CMC2/HCST/PELI1/C5AR1/SH3BGRL3/TLE4/RP2/TIMM23/YPEL3/GMFG/IRF2/GLRX/IRF9/XRN1/NCF4/HLA-DRB5/HCAR3/LYN/LRRK2/PTMS/XAF1/PHF11/ACTB/GNLY/STEAP4/ZNF385A/TPP1/IFI44/CCL2/MIER1/USP15/SP140L/SF3A2/RAB8A/CYSTM1/CST7/TRIM21/CD48/PSENEN/PLBD1/BCL3/FAM8A1/FCGR2A/IFIH1/DMBT1/CXCL16/UBE2L6/ALPK1/STAT4/CYBB/SNORA48/PI4K2B/STRN4/BIN2/ACSL1/TAOK3/ARHGDIB/CERS6/NFKBID/LMNB1/GK/CSF2RB/CASP4/TRIM22/HBB/TNIP3/GPR65/STAT1/JUND/VNN3/MPV17/HERC6/HCAR2/FPR2/ZC3HAV1/IL1B/HIST1H2BD/CHIT1/TNFAIP6/REPS2/SERPINA1/GBP4/MMP25/LGALS9/CD38/PCGF5/GCA/MCTP2/LILRB3/ANXA2R/ALPL/CA12/LTF/POU2AF1/GNG5/C1QC/CCL18/SAA1/DBNDD1/GBP1P1/SLAMF7/PILRA/ITGB2-AS1/TLR1/CMPK2/ARL5B/F8A1/CCDC114/PIK3AP1/LILRB2/OR7E14P/RGL4/TRPA1/RAC2/CD69/CTSE/IFIT5/LST1/IL1A/GNG2/IL1R2/RNF19B/PTK2B/LCN2/GBP1/CCR1/DDX58/BLOC1S1/FGL2/UCP2/SP110/CKLF/CXCL17/OASL/PATL1/C19orf66/FGR/PROK2/SAMSN1/CLEC7A/LOC399715/BASP1/LCP1/SLC30A4/P2RY14/IFITM3/CXCL11/RTP4/EVI2B/LAMP3/CYP7B1/HIST2H2BE/S100P/TMEM154/PLAC8/C3AR1/VSIG10L/ARRB2/ALDH3A1/S100A11/SPI1/DNAAF3/GINS4/TRIM38/S100A9/AIM2/PSTPIP2/RBP4/C15orf48/LCP2/APBB1IP/KLHDC7B/TYROBP/HRASLS2/FREM2/ZSWIM8-AS1/HIST1H2BC/DOK3/LRFN1/EPSTI1/GCH1/TNFSF14/EYA1/MARCKS/TNFSF10/HBA2/LBP/MGAM/GBP3/RSAD2/TREM1/CD53/PTPRZ1/TMEM71/CCL19/CDH2/IDO1/LILRA5/GCNT3/CTF1/KLK10/SERPINB9/CD37/LGALS17A/FCN1/CSF3R/CLEC4A/NBN/FCRLA/MX1/HSPB6/MNDA/SCN2A/IFITM1/F5/IFI44L/SPRR2E/CSTA/FPR1/ZBP1/SIRPB1/MXD1/CARD16/TNF/ST6GAL2/CHI3L1/CEACAM1/RGS18/HSH2D/HBA1/PADI2/ATP7B/MGC12916/SRGN/HERC5/CHI3L2/FBN2/LINC00605/CLDN2/TPSB2/MND1/ISG15/PPP2R2C/KCNJ2/S100A8/GBP5/WAS/EFCAB10/DDX60L/CDA/ANKRD22/HLA-J/FGL1/SAMD9L/CCL3/SYN1/BCL2L14/SAMD9/MAP2K6/CXCL10/CDHR5/FCGR3B/DNASE1/OAS2/TFEC/LRFN3/RHOH/MX2/CLCA3P/LY96/AQP9/TMEM171/SP140/IFIT3/TNFSF13B/GTSF1/LYPD1/IGSF6/RPH3A/IFIT2/EIF2AK2/DAPP1/ALOX5AP/LILRA6/METTL7B/GPR84/IL1RN/UPK2/OAS3/KCNJ2-AS1/BCL2A1/CCL11/GPX2/CYP4F3/IFITM2/CACNA1A/ZNF497/OAS1/IFIT1/OLR1/PLEK/TCN1/BCDIN3D-AS1/SIGLEC14/CDR1/FCER1G/IFI6/APOBEC3A/GPR141/IL2RG/CCL8/S100A12/CLEC4D/TM4SF20/FFAR2/IFNL1/ARSE/VWA7/IL17C/CLEC4E/KRT81/P2RY13/CCL4/ST20/CARD17/CPLX2/MUM1L1/TRPM8/SELL/AKR1B10/GABRA5/FLG/FDCSP/CRCT1 | |
|  | Down-regulated | CLEC3B/RRAD/SMTN/ARRDC2/PHLDB1/CA4/MAP3K6/GAS6/S100A13/PLXNA2/GPIHBP1/DOCK6/TCF25/ARHGEF15/CYP3A5/PER1/ITGB3/LMOD1/THOC6/GAK/WISP2/NOTCH4/KCNK3/CD82/PRELID1/TNXB/SEMA3F/ARAP3/SLC25A44/TBCD/S100A2/PPP1R14B/GRPEL2/BAP1/GPR146/MAFG/ATG4B/ACTR1B/KCTD20/GUCD1/RAPGEF3/HIF3A/FBXW5/INPP5A/TCERG1/SFRP2/PIK3IP1/TXNDC11/FZD4/EDC4/PPP1R13B/CBX7/TRAK1/SLC25A4/LMBR1L/FENDRR/PYCR2/RXRB/MOAP1/XAB2/DCHS1/LCN10/RNMT/TBX2/MFNG/STARD10/GAL/GATA2/SLC25A29/TRIM29/PQLC1/RASL12/TFIP11/43892/MICAL3/C2CD2/PCID2/NT5DC2/SPSB3/CRY2/SGSM2/MADD/ABTB1/BCOR/ZFAND2B/NOD1/ZBTB5/FAM160B2/PRKAB1/ITPR1/ZSWIM8/ZNF451/ARHGAP10/MYO1D/ISYNA1/PLEKHM1/FAM118A/ELK3/RSAD1/DTX4/PODN/PPP6R2/MFAP1/OTUD3/AKAP17A/HMGN3/ENTPD6/XPC/ADCY6/DAAM2/PPP1R3C/PRG2/CD320/SPRED1/CDKN2AIP/ASB1/DENND2A/PGF/NFATC4/SH3PXD2B/F2RL3/TMTC1/NR1D2/TSPAN18/SKIV2L/CC2D1B/NCAPH2/ADAMTS2/ITM2A/EIF4E2/NEBL/LZTR1/CTR9/ZSCAN18/PPP1R16B/SGSM3/SLC9A1/GLT8D1/GCHFR/RNF31/TSR1/LINC00312/ITPA/ATRX/PRKY/KRT17/CHFR/ULK3/METTL3/FUT1/LETM1/EXOSC7/CTCF/C19orf48/TMEM255B/ENDOD1/GPD1L/TTC7A/RASD2/SULT1A1/LDB1/THAP2/DYRK3/SMPD4/MLXIP/MPHOSPH10/SNX11/CCDC130/PLLP/HARS2/EDC3/MZT2B/DLL1/MEIS1/MBIP/WDR37/ZNF160/TAF1/GADD45G/MRPL11/TECPR1/SEPHS1/USP19/MDC1/LRRC47/URB1/SHE/MRPL55/STARD9/STUB1/ICAM2/PITPNB/VPS54/RAI2/ARHGEF25/CXorf40B/CAMK1/HSPA12B/SEMA6B/TEX264/SGCA/MTMR9LP/GMPPA/UCK1/PAN2/SFTA3/KAT5/TAZ/OSBPL5/ILVBL/SESN2/ATP6V0A2/NAB2/AKAP1/ASMTL/DHX29/ZBTB44/BAALC/MLH1/NFATC2/PYGO2/ATAD3B/ME3/ASAP2/CAB39L/RMDN1/S1PR2/RGS12/KCTD15/MEG3/KHDRBS3/ACP2/NOL8/KLHL42/ARHGAP4/DEXI/VSTM4/ICAM4/ZNF76/VSIG2/MYZAP/DTX3/TMEM88/FAM13B/RPS6KB2/MUM1/C14orf132/CALHM2/HPS4/EMC9/NDNF/ZBTB10/DAAM1/NMRAL1/LDLRAP1/PPTC7/PJA1/ZHX3/STAP2/TRAF5/ACTL6A/PER3/TMEM141/PPM1M/CLN3/FLYWCH2/PTK7/TRPC6/RAB4A/THAP7/OSGEP/GRAP/PALM/ITSN1/R3HCC1/TBX4/SMAD1/ORAI2/PHLDA3/NFRKB/SIDT2/DPH1/TMEM160/SNED1/CCDC84/BANP/B4GALT2/CCDC137/COL16A1/ACVR1/PANK4/CCDC85C/FAM199X/CWF19L2/TSFM/ZNF3/KCNAB2/TMEM243/UBIAD1/DNAJC18/SLC22A23/NSUN4/PDE9A/FLYWCH1/TRMT61A/SLC4A1AP/AGO1/MTIF3/DHDDS/IFFO1/CLCN6/RAB11FIP3/REST/DDB2/PLEKHG3/SSBP4/ZBTB47/GKN2/NEIL2/SLC35B2/CTDP1/RNF144A/RIOK2/C1orf216/ECSIT/CLCN7/NELFA/UPK3B/CPAMD8/WDR91/TTC14/MTX1/SERGEF/ARC/MPST/ESF1/TMEM39B/AMBRA1/COG1/SRSF10/MPLKIP/TMEM125/CACNA1C/TRIT1/STK19/METTL23/VPS8/ATAD3A/SLC35F2/RFXANK/AEBP2/FAM53B/ANKRD54/PTBP2/EGR2/MLH3/ACSS1/PTDSS2/SLC6A8/DHX32/KCTD11/SEMA6D/ARMC6/SNX21/CEP250/KLHL3/SNHG9/ACAD8/CD8A/ITGA10/PIN4/GFER/TPT1-AS1/RNF168/GPRC5B/ISY1/TOE1/DGKE/NAA35/ING5/PRKCZ/GCFC2/SNX25/CUL9/PLAGL1/SNORA70/MAP2K7/TRMU/ST3GAL3/PIAS2/ATP10A/MICALL2/LIN37/TBP/AFG3L1P/CCDC22/SNRNP35/GABRE/DTX2/SFRP4/XYLT1/DENND6A/AFMID/ANKRD29/KIAA0513/FAM174B/ZCCHC7/SNAPC2/ASB8/PARP2/CBFA2T2/DCAF16/PIK3R4/ZNF513/GPN2/HSDL1/FLJ23867/ENKD1/APC/RASSF1/SFN/NDST1/MMP1/TUBA4A/CRIP1/ADAM15/PLEKHM2/BCR/PDGFRB/SPHK1/FOS/MCM7/WDR74/CXorf36/PDE2A/EPHB4/SREK1/PRX/KLHDC3/ZC3H7A/MMP10/CYP1A1/BLCAP/SIN3B/HBP1/MCF2L/ACVRL1/SLC38A5/VWF/NDRG2/FOSB/NES/CTPS1/FBXO31/CLDN5/TSSC4/LRIG1/KANK2/FES/H19/PPIG/KANK3/CD34/ERRFI1/ST6GALNAC6/ENO2/ROBO3/ITPR3/GET4/ASL/RASA3/TUBB4B/CAMTA2/ITPKB/HCFC1R1/FAM214B/NR2C2/ADNP2/RALGDS/INMT/PTCD3/TTL/PCDH12/TBC1D9/KIAA0930/MTHFR/STC1/ATF6B/IFT43/SURF1/RARRES2/SLC25A36/SLC25A25/MIR5047/PHF13/ADORA2A/ATP8B2/MYO9B/MAPKBP1/MUL1/C12orf57/OTUD7B/DNAJB4/PODXL/SMAD7/CYTH1/NLRP1/INPP5K/ZCCHC24/MAF1/HSPA2/ITPKC/EHMT1/TSC1/SSNA1/REV3L/SIVA1/BTNL9/C6orf106/MICAL1/PDRG1/FASTK/SLC41A3/H2AFJ/FAM32A/HIRA/COL12A1/SH3BP5/COG4/KCTD3/POLR1E/MSC/CCT8/NF2/RPRD1A/PRPF39/CCNL2/VPS13D/KRI1/GALNT18/RFC1/SMC3/CWC22/PLXNB1/FIBP/TMEM208/TRAF2/RANBP10/PPP2R5B/BTN2A2/ISG20L2/PXDN/PEX5/SPRYD3/DNAJC7/SELENBP1/SSH2/UROD/EIF2B4/RAF1/PTGIS/RALBP1/SLC39A7/ZMYM2/WIPI1/UBE2J2/CANT1/PLAC9/BAIAP2/ARHGEF7/TMEM51/VPS37B/NME3/KIF3B/TPCN1/TFPI2/PPP3R1/PALD1/KDM1A/DLST/BAIAP2L1/LUZP1/TCF21/DNMT1/ANKZF1/UPP1/GGA2/ARFRP1/SPG7/CENPT/AGAP3/QARS/CHMP1A/B4GALT3/KCTD2/GNL1/FASTKD2/ELAC2/NR4A1/CD2BP2/ZGPAT/AMFR/UTP6/CKB/CSPG4/ADCY4/GGA3/JUN/TGFBR1/USP40/LCN6/PMM2/SH3PXD2A/PUS1/EIF2B5/SNAI1/TNFAIP1/ODC1/ZDHHC7/ZNF330/NCALD/MAFF/BRD9/CUL1/MYCT1/GOLGA8A/RND1/HLA-H/AQP1/MMP14/SPARCL1/RBM19/TRAK2/EPC1/CDC37/SASH1/ATP6V0A1/WBP1L/MFGE8/URGCP/BSDC1/ARHGAP29/ERCC1/FARP1/CDK9/PXDC1/TMEM109/MAPKAPK2/EBNA1BP2/RASIP1/HEXA/TANC1/GNL2/RBFOX2/MCAM/RHBDF1/TSPYL2/PIEZO1/DPP7/AEN/HDAC3/CIB1/TACC1/PSMC5/UNC13B/TRPC4AP/NOP56/CIRBP/TUBGCP2/RGS3/GIMAP6/TSPAN4/SGTA/IER2/CCND1/BCAR1/KLF13/EGR1/TNS1/ENG/SEMA4C/FDPS/TUBA1C/SLC35A4/DDX42/HERPUD1/VPS28/NOTCH2/GRB10/ABLIM1/CTTN/AHNAK/ROBO4/ANAPC11/PRMT1/HDAC7/PDK4/USF1/APH1A/DDX39A/RPL3/RPSA/PLXND1/RPS6KA2/MAT2A/DST/TMBIM1/MAP4/PSMD2/ARPC1B/NDRG1/SERTAD1/MGLL/A2M/SERPINB6/XRCC6/LMNA/HMOX1/SSH1/QSOX1/TGFBR2/RPS28/RPLP0 |  |
| GSE150316 | Up-regulated | ZNF557/SLC22A17/NSDHL/LMAN2L/SNRPD2/RARG/TTC5/ABCA10/NBPF11/SCEL/ZNF813/CCDC77/LSM7/FAM46A/GEMIN6/MKKS/ZC3HAV1/ATP8B4/PSPH/CXorf36/JAM2/CADM1/ATP13A4/SNORA73B/THSD7A/MYEOV2/APOBEC3G/GHDC/AVIL/PSD4/BST2/TEC/CRYZ/SHANK2/MED18/RASSF9/AJUBA/GNAL/CLU/TOMM5/KIFAP3/PRRG4/SCAI/SELK/P4HA2/ITGB3BP/STAT4/PRICKLE4/TRIM69/ELP5/TTLL7/ZNF410/MISP/MS4A14/THYN1/TAP1/NPIPB11/NOSTRIN/FANCL/IL32/FAM120C/SAMD9/RHPN2/CIITA/SNORD3C/VILL/SNORD89/TNFSF10/TDP1/XAF1/ELOVL7/PRR5L/ACYP2/FLRT3/CLUHP3/SLC15A2/RASGRP3/SNORA74B/CTD-2020K17.1/IL3RA/IFI35/RFC5/DNM3OS/SIDT1/SELP/APOBEC3F/COX7A1/CETN3/SCNN1B/MMP28/TYSND1/CRIP1/NBPF1/FOLR1/LRRC27/CHN2/C11orf63/ZNF702P/PHGDH/ARNT2/IFT43/SNORA55/CFAP54/AL139099.2/C17orf75/BCL11B/KIF15/RNA5-8S5/C1GALT1C1/RN7SL396P/ZWILCH/ZNF793/CCDC171/PDE10A/DOLK/CCDC167/RNU12/THUMPD3-AS1/ZBTB7C/FGFR1OP/NHS/IFIH1/PIGP/PSMD10/RARRES1/ENPP5/3-Mar/RAMP2/KIAA0825/FOXP2/TRIM16/SNORA58/HIST1H2AG/HDAC9/GABRE/DHRS7B/HMCN2/PLVAP/SNHG11/TIFA/SNORA79/MGST2/SRP19/MB21D1/RARRES3/AL355075.1/PLLP/NLRP2/SNORD116-15/FRZB/CD2/HIBCH/C2orf88/BUB1/HIST1H1B/PHYHD1/PROSER2/HERC2P3/ZNF382/SNORA45B/SAMD9L/AMOT/IFI27L2/SNORD116-1/PIK3CG/SIRT5/1-Sep/STYXL1/OASL/HIST1H3H/CTA-414D7.1/RN7SL38P/RAB17/RPL23AP82/CCDC85A/ITGA2B/CA11/GATB/DDX58/TIAF1/METTL12/C11orf84/KAL1/FLRT2/DNAJA1/PLCXD3/LINC00493/STIP1/SP140/SNORA65/CKS2/TMEM19/ADAMTS6/CFB/BANK1/CD28/ADAM28/IFITM1/PAX8-AS1/APOBEC3D/ITM2C/CAPN13/ZNF519/SNORA33/ABCB1/RFTN2/SNORA71D/SNORA11/IRF4/SNORA5C/KL/AP4B1/SPTLC3/NOVA1/CYP51A1/HIST1H4I/AC007743.1/CCSER1/CILP/BATF2/NDUFA9/HAUS8/HIST1H2BN/TNNT2/FCRL5/SNORD116-8/AKAP2/RNVU1-19/LAX1/SNORA5A/LL22NC03-86G7.1/COL8A2/IGF2/RPA3/RP11-329L6.2/PSMB10/ICAM2/FRRS1/UBBP4/SNORD116-6/AVPR1A/MGAT3/C12orf75/SNORD46/SEMA3E/HIST1H2AH/HAPLN3/KIF2C/CCNB1/RP5-1039K5.19/RNU6ATAC/CUBN/IGKV3-15/CCL19/KCNN3/MS4A1/TLR7/EML6/MFAP3L/SLC19A3/CLEC3B/HSH2D/SGSM1/HSPA7/FAM98C/RNVU1-10/SLAMF1/DEPDC1B/EFR3B/MUC3A/IGKV3-11/IGHV3-49/RNU1-1/IGKC/NPTXR/CACYBP/REEP1/THAP2/RAMP3/SNORD71/IGHA2/CDON/IGLV2-14/ISG15/IL17RD/NRXN3/TICRR/IGHG2/TRIM29/ZBP1/HIST1H2BL/RNVU1-7/IGHV3-15/FSIP2/ZBED2/IGHG1/SNORA40/LNX1/IGHV3-53/NAALAD2/HIST1H2BF/PLA2G2A/AL353644.10/RBM20/IGKV1-5/CTD-2017F17.2/IGKV1-9/SDR42E1/TP63/HIST1H2BO/RNVU1-18/AC008079.10/IGJ/KIAA0125/IGHV3-23/RTKN2/KIAA1324/IGHA1/HIST1H3C/ACKR1/SNORA35/ANKRD36BP2/ADCY1/FAT2/TFAP2A/FGF10/IGKV1-16/IGLV2-11/FOLR2/CYP2J2/PPBP/GZMA/CTA-228A9.4/IGLV1-44/HIST1H3G/IDO1/ZFHX4/IGHV4-39/AQP7/SNORD116-2/POU2AF1/APOL4/IGHV3-21/IGLC2/IGHGP/GBP1P1/SRL/FNDC1/GPM6A/PTGER3/CHRM3/IGLV1-51/ADAMTSL1/IGKV4-1/IGHV3-30/IGHV3-33/KIF5C/ELOVL2/MZB1/CCL8/XIRP2/MAPT/IGHG4/DSC3/IGHV1-18/IGLV2-23/IGLC3/IGHV5-51/KIAA1644/FLG/PRLR/IGLL5/RNU1-28P/RGMA/IGLV3-19/IGHV1-2/RP11-217B1.2/IGLV1-40/IGHG3/PF4/IGKV1-39/S100A2/SNORA13/LAG3/IGHV3-74/IGHM/LTF/IGLV3-1/HSP90AA2P/CLCA2/TENM2/GRIP1/PCDH10/IGHD/MCF2L2/MTUS2/IGLV1-47/IGLV3-10/ACTC1/SYT1/MYO18B/IGLV3-25/SLC5A1/SNORA38B/MMP11/IGHV1-69-2/IGHV1-46/GRIN2B/PCK1/APLNR/HELLPAR/STRA6/IGLV9-49/TF/IGHV2-70/IGLV3-21/IGHV4-34/BPIFB1/IGHV4-59/PPP1R1A/MYLK3/NRXN1/MIR205HG/MYOM3/HES2/C10orf71/DUX4L26/IGLV4-69/RP3-323A16.1/MLIP/FBXO40/TCL6/EMX2OS/AC006548.28/MUC17/PRB4/CTA-796E4.5/ORF10/ORF7b/E/ORF8/ORF6/ORF7a/ORF3a/M/N/S/ORF1b/ORF1a |  |
|  | Down-regulated | MTND1P23/SERPINB2/SLC16A6/GDF15/AC068580.6/GADD45A/RP11-750B16.1/AC007278.3/MMP1/RP1-309I22.2/EGR1/RP11-417F21.1/POSTN/SLC7A11/IGFN1/HILPDA/NR4A1/PI15/CXCL8/RBM3/CCL18/CYR61/NUDT16/FUCA1/CCL20/GLDN/ZNF638-IT1/MT-CO3/SPDYA/CYGB/MT-ATP6/ZFP36/NPL/MT-ND4/APOLD1/CH507-42P11.8/MTATP6P1/ADM/MIR143HG/HSPB7/MT-CO2/IER3/PLA2G7/ESM1/RP11-244F12.3/BCAT1/MT-CO1/MT-ATP8/MT-ND2/MPEG1/MYL3/MTND4P12/HSPB6/C5AR1/GPR97/MTND2P28/NME9/SLC19A2/UPP1/USP53/RASD1/GREM1/IL1R2/APLN/LACC1/KLF9/GLUL/NR1D1/HK2/FLJ42393/RP11-415J8.3/CTD-2033D15.2/EHBP1L1/ANKRD37/AC093838.4/RP13-20L14.10/MT-CYB/SIGLEC14/PTGS2/RP11-563J2.2/PLIN2/MT-ND6/C10orf10/CD300A/RP11-437B10.1/BHLHE40/RRAD/MMP19/ERRFI1/PER1/SLED1/RP11-93B14.9/MAPK6/SLC6A6/MAFK/MT-ND1/RRM2B/ALOX15B/MARCO/NKD2/ZFAND5/SCARB1/MT-ND5/SLC6A14/TCF21/BHLHE41/INSIG1/RNF152/SLCO4C1/SLC2A3/CYP1B1/CSF3R/MKRN5P/MDM2/ADAMTS1/CD300E/FOSL1/GK/RCN1P2/CSGALNACT2/ANKRD1/GPNMB/RP11-423E7.2/RHOQ/MT-ND4L/RASGEF1B/CSPG4/ACADSB/CCNG1/RP11-803D5.1/GPCPD1/KLF6/PLAU/DUSP5/MUC4/IL1RL1/RNF126/MT1X/FKBP5/DAAM2/SIGLEC10/PAG1/GUCY1A2/MSR1/MT-ND3/RLIM/TCEAL4/LILRB3/SLC11A1/ZMAT3/PRRT2/TFRC/UAP1L1/JUNB/RPGR/PTPN18/SLCO4A1/RP11-463O12.5/PLBD2/SNTB1/LAMC3/ID4/IRAK3/MME/COL6A6/YJEFN3/TXNRD1/PPP1R3B/NID1/ATP13A3/AL133243.1/RHOB/ATF3/PHLDA1/PDK4/FAP/FAM213B/ADAMTS2/AC092881.1/HMOX1/PTP4A1/ARAP1-AS2/RGL3/CTSK/LIPA/SLC7A8/AKAP12/CREG1/NCR3LG1/ITGAX/EIF4B/PTEN/ZNF224/GNPDA1 |  |
| The intersection gene |  | CCL8/SP140/ISG15/SAMD9L/SAMD9/IFITM1/RRAD/SIGLEC14/TNFSF10/HSH2D/CSF3R/CCL19/CRIP1/CLEC3B/IDO1/FLG/DDX58/ERRFI1/MMP1/ZBP1/HSPB6/OASL/ZC3HAV1/PER1/CCL18/LILRB3/EGR1/LTF/XAF1/GBP1P1/NR4A1/STAT4/ADAMTS2/GK/TCF21/UPP1/S100A2/C5AR1/IFIH1/DAAM2/IL1R2/CSPG4/THAP2/IFT43/CXorf36/PLLP/POU2AF1/ICAM2/TRIM29/HMOX1/PDK4/GABRE. |  |
