# Supplementary material for: An integrative bioinformatics analysis for identifying hub genes associated with infection of lung samples in patients infected with SARS-CoV-2
Source: Eur J Med Res. 2021 Dec 17;26:146. doi: 10.1186/s40001-021-00609-4 (PMC8677925; doi:10.1186/s40001-021-00609-4)
Supplement: Supplementary file 3 — Additional file 3: Table S2. The function and fold change of 52 identified genes. [file 40001_2021_609_MOESM3_ESM.docx]

**Supplementary Table S2. The function and fold change of 52 identified genes.**

| ID | Function | logFC |
| --- | --- | --- |
| CCL8 | Displaying chemotactic activity for monocytes, lymphocytes, basophils and eosinophils. | 3.05166989 |
| SP140 | Variants of this gene have been associated with multiple sclerosis, Crohn's disease, and chronic lymphocytic leukemia. | 1.62320805 |
| ISG15 | Including chemotactic activity towards neutrophils, direction of ligated target proteins to intermediate filaments, cell-to-cell signaling, and antiviral activity during viral infections. | 2.22792718 |
| SAMD9L | Encoding a cytoplasmic protein that acts as a tumor suppressor but also plays a key role in cell proliferation and the innate immune response to viral infection. | 1.53285197 |
| SAMD9 | Encoding a sterile alpha motif domain-containing protein that may play a role in regulating cell proliferation and apoptosis. | 1.18214997 |
| IFITM1 | Involving Interferon gamma signaling and Immunoregulatory interactions between a Lymphoid and a non-Lymphoid cell. | 1.67951461 |
| TNFSF10 | Encoding a cytokine that preferentially induces apoptosis in transformed and tumor cells, but does not appear to kill normal cells. | 1.19912603 |
| HSH2D | Diseases associated with HSH2D include Parietal Foramina. | 2.05234667 |
| CCL19 | Playing a role in normal lymphocyte recirculation and homing, in trafficking of T cells in thymus, and in T cell and B cell migration to secondary lymphoid organs. | 2.00728459 |
| CRIP1 | It may be involved in intestinal zinc transport. | 1.27056462 |
| CLEC3B | Involving pathways that are response to elevated platelet cytosolic Ca2+. | 2.05080735 |
| IDO1 | Encoding indoleamine 2,3-dioxygenase (IDO) that plays a role in a variety of pathophysiological processes such as antimicrobial and antitumor defense, immunoregulation, and antioxidant activity. | 2.66118974 |
| FLG | Encoding an intermediate filament-associated protein that aggregates keratin intermediate filaments in mammalian epidermis. Mutations in this gene are associated with ichthyosis vulgaris. | 3.15987816 |
| DDX58 | Involving in viral double-stranded (ds) RNA recognition and the regulation of the antiviral innate immune response. | 1.58716501 |
| ZBP1 | Encoding a Z-DNA binding protein that plays a role in the innate immune response by binding to foreign DNA and inducing type-I interferon production. | 2.28518362 |
| OASL | Diseases associated with OASL include West Nile Fever and West Nile Virus Infection. | 1.55560858 |
| ZC3HAV1 | Encoding a CCCH-type zinc finger protein that may primarily function to inhibit viral gene expression and induce an innate immunity to viral infection. | 1.04197012 |
| LTF | Encoding the protein that demonstrates a broad spectrum of properties, including regulation of iron homeostasis, host defense against a broad range of microbial infections, anti-inflammatory activity, regulation of cellular growth and differentiation and protection against cancer development and metastasis. | 3.47830783 |
| XAF1 | Encoding a protein which binds to and counteracts the inhibitory effect of a member of the IAP (inhibitor of apoptosis) protein family. (IAP proteins bind to and inhibit caspases which are activated during apoptosis). | 1.20294929 |
| GBP1P1 | It is a pseudogene. | 2.78737712 |
| STAT4 | Encoding the protein that is essential for mediating responses to IL12 in lymphocytes, and regulating the differentiation of T helper cells. | 1.12373159 |
| S100A2 | Encoding the protein which involved in the regulation of a number of cellular processes such as cell cycle progression and differentiation and may have a tumor suppressor function. | 3.32256108 |
| IFIH1 | Encoding MDA5 which is an intracellular sensor of viral RNA that triggers the innate immune response, involves in a proinflammatory response that includes interferons and plays an important role in enhancing natural killer cell function in malaria infection. | 1.38917091 |
| THAP2 | It may involve in nucleic acid binding. | 2.15254866 |
| IFT43 | Encoding a subunit of the intraflagellar transport complex A (IFT-A) which plays an important role in cilia assembly and maintenance by mediating retrograde ciliary transport. | 1.31674814 |
| CXorf36 | Diseases associated with it include Autism and Ischiocoxopodopatellar Syndrome With Or Without Pulmonary Arterial Hypertension. | 1.05248573 |
| PLLP | Diseases associated with it include Bardet-Biedl Syndrome and Neuropathy, Congenital Hypomyelinating, 1, Autosomal Recessive. | 1.47701512 |
| POU2AF1 | Diseases associated with POU2AF1 include Hodgkin's Lymphoma, Lymphocytic Depletion and Primary Biliary Cholangitis. | 2.69626759 |
| ICAM2 | Encoding the protein which may play a role in lymphocyte recirculation by blocking LFA-1-dependent cell adhesion. | 1.92561727 |
| TRIM29 | Encoding the protein that may act as a transcriptional regulatory factor involved in carcinogenesis and/or differentiation and may also function in the suppression of radiosensitivity | 2.27379043 |
| GABRE | Encoding the gamma-aminobutyric acid (GABA) which is a multisubunit chloride channel that mediates the fastest inhibitory synaptic transmission in the central nervous system. | 1.44015853 |
| RRAD | Diseases associated with it include Benign Pleural Mesothelioma and Type 2 Diabetes Mellitus. | -1.25576521 |
| SIGLEC14 | It is a protein coding gene. Among its related pathways are Innate Immune System and RET signaling. | -1.30076177 |
| HMOX1 | It is an essential enzyme in heme catabolism, cleaves heme to form biliverdin, which is subsequently converted to bilirubin by biliverdin reductase, and carbon monoxide, a putative neurotransmitter. | -1.01950078 |
| PDK4 | Encoding a mitochondrial protein which inhibits the pyruvate dehydrogenase complex by phosphorylating one of its subunits, thereby contributing to the regulation of glucose metabolism. | -1.03428653 |
| DAAM2 | Diseases associated with DAAM2 include Nephrotic Syndrome, Type 24 and Idiopathic Steroid-Resistant Nephrotic Syndrome. | -1.10188134 |
| IL1R2 | Encoding the protein which binds interleukin alpha (IL1A), interleukin beta (IL1B), and interleukin 1 receptor, type I(IL1R1/IL1RA), and acts as a decoy receptor that inhibits the activity of its ligands. | -1.36565181 |
| CSPG4 | It plays a role in stabilizing cell-substratum interactions during early events of melanoma cell spreading on endothelial basement membranes. | -1.14066765 |
| ADAMTS2 | Mutations in this gene cause Ehlers-Danlos syndrome type VIIC, a recessively inherited connective-tissue disorder. | -1.02199829 |
| GK | Encoding the protein which is a key enzyme in the regulation of glycerol uptake and metabolism. | -1.15274467 |
| TCF21 | Encoding a transcription factor of the basic helix-loop-helix family which is mesoderm specific, and expressed in embryonic epicardium, mesenchyme-derived tissues of lung, gut, gonad, and both mesenchymal and glomerular epithelial cells in the kidney. | -1.18989589 |
| UPP1 | Encoding the enzyme which functions in the degradation and salvage of pyrimidine ribonucleosides. | -1.39160241 |
| PER1 | Encoding components of the circadian rhythms of locomotor activity, metabolism, and behavior. Polymorphisms in this gene may increase the risk of getting certain cancers. | -1.24760541 |
| CCL18 | Encoding the cytokine which displays chemotactic activity for naive T cells, CD4+ and CD8+ T cells and nonactivated lymphocytes. And it may play a role in both humoral and cell-mediated immunity responses. | -1.70069555 |
| LILRB3 | Encoding the protein which is thought to control inflammatory responses and cytotoxicity to help focus the immune response and limit autoreactivity. | -1.07653388 |
| EGR1 | Encoding a nuclear protein which functions as a transcriptional regulator. | -2.01699615 |
| CSF3R | Encoding the protein which may function in some cell surface adhesion or recognition processes. | -1.17365562 |
| ERRFI1 | It is induced during cell stress and mediates cell signaling. | -1.25233948 |
| MMP1 | Encoding the protein which is involved in the breakdown of extracellular matrix in normal physiological processes, such as embryonic development, reproduction, and tissue remodeling, as well as in disease processes, such as arthritis and metastasis. | -2.14592369 |
| HSPB6 | Encoding the protein which likely plays a role in smooth muscle relaxation. | -1.41548362 |
| NR4A1 | Encoding the protein which acts as a nuclear transcription factor. And translocation of the protein from the nucleus to mitochondria induces apoptosis. | -1.74283470 |
| C5AR1 | Diseases associated with C5AR1 include Hypersensitivity Reaction Type Iii Disease and Mast-Cell Sarcoma. | -1.41359835 |
