# Supplementary material for: An integrative bioinformatics analysis for identifying hub genes associated with infection of lung samples in patients infected with SARS-CoV-2
Source: Eur J Med Res. 2021 Dec 17;26:146. doi: 10.1186/s40001-021-00609-4 (PMC8677925; doi:10.1186/s40001-021-00609-4)
Supplement: Supplementary file 4 — Additional file 4: Table S3. BP pathways of GSE147507 and GSE150316 that are heavily enriched in GO analysis (ranked in the top 10 according to p value). [file 40001_2021_609_MOESM4_ESM.docx]

**Supplementary Table S3. BP pathways of GSE147507 and GSE150316 that are heavily enriched in GO analysis (ranked in the top 10 according to P value).**

| The data set | ID | Description | Count | P-value | GeneID |
| --- | --- | --- | --- | --- | --- |
| GSE147507 | GO:0009615 | response to virus | 56 | 6.63407617950802E-15 | IFI6/CCL8/CCL4/IFIT1/OAS1/IFITM2/CCL11/IFIT3/EIF2AK2/IFIT2/APOBEC3A/MX2/OAS3/ISG15/OAS2/MX1/IFITM1/HERC5/IFI44L/TRIM38/CXCL10/CCL19/IFITM3/TNF/FGR/DDX58/RSAD2/IFIT5/LGALS9/OASL/GBP3/ZC3HAV1/RTP4/FGL2/BCL3/LCN2/GBP1/STAT1/TRIM22/ODC1/IRF9/IFI44/CYP1A1/CFL1/IRF2/IFIH1/CCDC130/DMBT1/IFNL1/PARP9/IL1B/PIM2/MUL1/AIM2/IRF7/STAT2 |
|  | GO:0060337 | type I interferon signaling pathway | 28 | 4.95902534356536E-14 | IFI6/IFIT1/OAS1/IFITM2/IFIT3/IFIT2/MX2/OAS3/ISG15/OAS2/MX1/IFITM1/IFITM3/RSAD2/ZBP1/OASL/CDC37/STAT1/EGR1/XAF1/IRF9/METTL3/IRF2/MUL1/IRF7/HLA-H/HLA-A/STAT2 |
|  | GO:0071357 | cellular response to type I interferon | 28 | 4.95902534356536E-14 | IFI6/IFIT1/OAS1/IFITM2/IFIT3/IFIT2/MX2/OAS3/ISG15/OAS2/MX1/IFITM1/IFITM3/RSAD2/ZBP1/OASL/CDC37/STAT1/EGR1/XAF1/IRF9/METTL3/IRF2/MUL1/IRF7/HLA-H/HLA-A/STAT2 |
|  | GO:0034340 | response to type I interferon | 28 | 1.56374889796681E-13 | IFI6/IFIT1/OAS1/IFITM2/IFIT3/IFIT2/MX2/OAS3/ISG15/OAS2/MX1/IFITM1/IFITM3/RSAD2/ZBP1/OASL/CDC37/STAT1/EGR1/XAF1/IRF9/METTL3/IRF2/MUL1/IRF7/HLA-H/HLA-A/STAT2 |
|  | GO:0002283 | neutrophil activation involved in immune response | 67 | 1.04209227016626E-12 | SELL/FCER1G/OLR1/CLEC4D/GPR84/S100A12/SIGLEC14/PADI2/TYROBP/S100A11/S100A8/CD53/FPR1/DOK3/CEACAM1/CHI3L1/S100P/CDA/TUBB4B/PLAC8/SIRPB1/FGR/MNDA/S100A9/C3AR1/FCGR3B/SERPINA1/FGL2/LILRB2/GCA/CYSTM1/TCN1/FCN1/LCN2/LILRB3/ACTR1B/CHIT1/CYBB/BCR/FCGR2A/LTF/TNFAIP6/MGAM/BIN2/CCT8/GMFG/C5AR1/DYNLT1/MMP25/CEACAM6/ATAD3B/TMBIM1/DNASE1/CANT1/KCNAB2/ATP6V0A1/XRCC6/PRG2/HBB/CD55/DPP7/FPR2/PSMD2/HLA-H/SERPINB6/PIGR/QSOX1 |
|  | GO:0043312 | neutrophil degranulation | 66 | 2.32921667848297E-12 | SELL/FCER1G/OLR1/CLEC4D/GPR84/S100A12/SIGLEC14/PADI2/TYROBP/S100A11/S100A8/CD53/FPR1/DOK3/CEACAM1/CHI3L1/S100P/CDA/TUBB4B/PLAC8/SIRPB1/FGR/MNDA/S100A9/C3AR1/FCGR3B/SERPINA1/FGL2/LILRB2/GCA/CYSTM1/TCN1/FCN1/LCN2/LILRB3/ACTR1B/CHIT1/CYBB/BCR/FCGR2A/LTF/TNFAIP6/MGAM/BIN2/CCT8/GMFG/C5AR1/DYNLT1/MMP25/CEACAM6/ATAD3B/TMBIM1/CANT1/KCNAB2/ATP6V0A1/XRCC6/PRG2/HBB/CD55/DPP7/FPR2/PSMD2/HLA-H/SERPINB6/PIGR/QSOX1 |
|  | GO:0042119 | neutrophil activation | 67 | 2.68216856945637E-12 | SELL/FCER1G/OLR1/CLEC4D/GPR84/S100A12/SIGLEC14/PADI2/TYROBP/S100A11/S100A8/CD53/FPR1/DOK3/CEACAM1/CHI3L1/S100P/CDA/TUBB4B/PLAC8/SIRPB1/FGR/MNDA/S100A9/C3AR1/FCGR3B/SERPINA1/FGL2/LILRB2/GCA/CYSTM1/TCN1/FCN1/LCN2/LILRB3/ACTR1B/CHIT1/CYBB/BCR/FCGR2A/LTF/TNFAIP6/MGAM/BIN2/CCT8/GMFG/C5AR1/DYNLT1/MMP25/CEACAM6/ATAD3B/TMBIM1/DNASE1/CANT1/KCNAB2/ATP6V0A1/XRCC6/PRG2/HBB/CD55/DPP7/FPR2/PSMD2/HLA-H/SERPINB6/PIGR/QSOX1 |
|  | GO:0002446 | neutrophil mediated immunity | 67 | 2.94297581580023E-12 | SELL/FCER1G/OLR1/CLEC4D/GPR84/S100A12/SIGLEC14/PADI2/TYROBP/S100A11/S100A8/CD53/FPR1/DOK3/CEACAM1/CHI3L1/S100P/CDA/TUBB4B/PLAC8/SIRPB1/FGR/MNDA/S100A9/C3AR1/FCGR3B/SERPINA1/FGL2/LILRB2/GCA/CYSTM1/TCN1/FCN1/LCN2/LILRB3/ACTR1B/CHIT1/CYBB/BCR/FCGR2A/LTF/TNFAIP6/MGAM/BIN2/CCT8/GMFG/C5AR1/DYNLT1/MMP25/CEACAM6/ATAD3B/TMBIM1/DNASE1/CANT1/KCNAB2/ATP6V0A1/XRCC6/PRG2/HBB/CD55/DPP7/FPR2/PSMD2/HLA-H/SERPINB6/PIGR/QSOX1 |
|  | GO:0034341 | response to interferon-gamma | 37 | 2.49585510817472E-11 | CCL8/CCL4/OAS1/IFITM2/CCL11/OAS3/OAS2/IFITM1/WAS/GCH1/TRIM38/GBP5/CCL19/IFITM3/CCL3/LGALS9/OASL/GBP3/CCL18/GBP4/CXCL16/CDC37/GBP1/STAT1/TRIM22/TRIM21/HLA-DRB5/IRF9/CCL2/HCK/IRF2/NMI/PARP9/IFI30/IRF7/HLA-H/HLA-A |
|  | GO:0051607 | defense response to virus | 41 | 2.64005712199736E-11 | IFI6/IFIT1/OAS1/IFITM2/IFIT3/EIF2AK2/IFIT2/APOBEC3A/MX2/OAS3/ISG15/OAS2/MX1/IFITM1/HERC5/IFI44L/TRIM38/CXCL10/IFITM3/DDX58/RSAD2/IFIT5/OASL/GBP3/ZC3HAV1/RTP4/FGL2/GBP1/STAT1/TRIM22/IRF9/IRF2/IFIH1/DMBT1/IFNL1/PARP9/IL1B/MUL1/AIM2/IRF7/STAT2 |
| GSE150316 | GO:0006958 | complement activation, classical pathway | 44 | 4.5197900223055E-36 | IGLV3-25/IGHV4-59/IGHG4/IGLL5/IGLV3-21/IGLV1-47/IGHV3-74/IGHV4-34/IGHV1-18/IGLV1-40/IGKV4-1/IGHV5-51/IGLC3/IGHM/IGHG3/IGLV1-44/IGKV1-39/IGLV1-51/IGHD/IGLC2/IGKV1-5/IGHA1/IGHV3-30/IGHV3-21/IGLV3-19/IGHV3-23/IGHG2/IGLV3-1/IGHV1-69-2/IGLV2-14/IGLV2-23/IGHV3-15/IGKC/IGHV4-39/IGHA2/IGHV3-33/CLU/IGKV1-16/IGLV2-11/IGHG1/IGKV3-15/IGHV2-70/IGHV3-53/IGHV3-49 |
|  | GO:0002455 | humoral immune response mediated by circulating immunoglobulin | 44 | 3.82919157894026E-34 | IGLV3-25/IGHV4-59/IGHG4/IGLL5/IGLV3-21/IGLV1-47/IGHV3-74/IGHV4-34/IGHV1-18/IGLV1-40/IGKV4-1/IGHV5-51/IGLC3/IGHM/IGHG3/IGLV1-44/IGKV1-39/IGLV1-51/IGHD/IGLC2/IGKV1-5/IGHA1/IGHV3-30/IGHV3-21/IGLV3-19/IGHV3-23/IGHG2/IGLV3-1/IGHV1-69-2/IGLV2-14/IGLV2-23/IGHV3-15/IGKC/IGHV4-39/IGHA2/IGHV3-33/CLU/IGKV1-16/IGLV2-11/IGHG1/IGKV3-15/IGHV2-70/IGHV3-53/IGHV3-49 |
|  | GO:0006956 | complement activation | 46 | 2.75955951999299E-33 | IGLV3-25/IGHV4-59/IGHG4/C5AR1/IGLL5/IGLV3-21/IGLV1-47/IGHV3-74/IGHV4-34/IGHV1-18/IGLV1-40/IGKV4-1/IGHV5-51/IGLC3/IGHM/IGHG3/IGLV1-44/IGKV1-39/IGLV1-51/IGHD/IGLC2/IGKV1-5/IGHA1/CFB/IGHV3-30/IGHV3-21/IGLV3-19/IGHV3-23/IGHG2/IGLV3-1/IGHV1-69-2/IGLV2-14/IGLV2-23/IGHV3-15/IGKC/IGHV4-39/IGHA2/IGHV3-33/CLU/IGKV1-16/IGLV2-11/IGHG1/IGKV3-15/IGHV2-70/IGHV3-53/IGHV3-49 |
|  | GO:0072376 | protein activation cascade | 46 | 1.07506942158653E-30 | IGLV3-25/IGHV4-59/IGHG4/C5AR1/IGLL5/IGLV3-21/IGLV1-47/IGHV3-74/IGHV4-34/IGHV1-18/IGLV1-40/IGKV4-1/IGHV5-51/IGLC3/IGHM/IGHG3/IGLV1-44/IGKV1-39/IGLV1-51/IGHD/IGLC2/IGKV1-5/IGHA1/CFB/IGHV3-30/IGHV3-21/IGLV3-19/IGHV3-23/IGHG2/IGLV3-1/IGHV1-69-2/IGLV2-14/IGLV2-23/IGHV3-15/IGKC/IGHV4-39/IGHA2/IGHV3-33/CLU/IGKV1-16/IGLV2-11/IGHG1/IGKV3-15/IGHV2-70/IGHV3-53/IGHV3-49 |
|  | GO:0006959 | humoral immune response | 57 | 9.08629547211735E-29 | IGLV3-25/IGHV4-59/IGHG4/C5AR1/IGLL5/IGLV3-21/IGLV1-47/IGHV3-74/IGHV4-34/IGHV1-18/IGLV1-40/IGKV4-1/IGHV5-51/POU2AF1/IGLC3/IGHM/PSMB10/IGHG3/IGLV1-44/IGKV1-39/BPIFB1/SLC11A1/IGLV1-51/IGHD/IGLC2/IGKV1-5/IGHA1/CFB/IGHV3-30/IGHV3-21/IGLV3-19/IGHV3-23/IGHG2/IGLV3-1/IGHV1-69-2/IGLV2-14/IGLV2-23/IGHV3-15/IGKC/PF4/IGHV4-39/PLA2G2A/IGHA2/IGHV3-33/LTF/CXCL8/CLU/IGKV1-16/IGLV2-11/IGHG1/CD28/IGKV3-15/IGHV2-70/IGHV3-53/PPBP/IGHV3-49/MS4A1 |
|  | GO:0016064 | immunoglobulin mediated immune response | 46 | 9.8751942308315E-29 | IGLV3-25/IGHV4-59/IGHG4/IGLL5/IGLV3-21/IGLV1-47/IGHV3-74/IGHV4-34/IGHV1-18/IGLV1-40/IGKV4-1/IGHV5-51/IGLC3/IGHM/IGHG3/IGLV1-44/IGKV1-39/IGLV1-51/IGHD/IGLC2/IGKV1-5/IGHA1/TFRC/IGHV3-30/IGHV3-21/IGLV3-19/IGHV3-23/IGHG2/IGLV3-1/IGHV1-69-2/IGLV2-14/IGLV2-23/IGHV3-15/IGKC/IGHV4-39/IGHA2/IGHV3-33/CLU/IGKV1-16/IGLV2-11/IGHG1/CD28/IGKV3-15/IGHV2-70/IGHV3-53/IGHV3-49 |
|  | GO:0019724 | B cell mediated immunity | 46 | 1.86012769572066E-28 | IGLV3-25/IGHV4-59/IGHG4/IGLL5/IGLV3-21/IGLV1-47/IGHV3-74/IGHV4-34/IGHV1-18/IGLV1-40/IGKV4-1/IGHV5-51/IGLC3/IGHM/IGHG3/IGLV1-44/IGKV1-39/IGLV1-51/IGHD/IGLC2/IGKV1-5/IGHA1/TFRC/IGHV3-30/IGHV3-21/IGLV3-19/IGHV3-23/IGHG2/IGLV3-1/IGHV1-69-2/IGLV2-14/IGLV2-23/IGHV3-15/IGKC/IGHV4-39/IGHA2/IGHV3-33/CLU/IGKV1-16/IGLV2-11/IGHG1/CD28/IGKV3-15/IGHV2-70/IGHV3-53/IGHV3-49 |
|  | GO:0030449 | regulation of complement activation | 34 | 9.76902799306495E-27 | IGLV3-25/IGHV4-59/IGHG4/C5AR1/IGLV3-21/IGLV1-47/IGHV4-34/IGLV1-40/IGKV4-1/IGLC3/IGHG3/IGLV1-44/IGKV1-39/IGLV1-51/IGLC2/IGKV1-5/CFB/IGHV3-30/IGLV3-19/IGHV3-23/IGHG2/IGLV3-1/IGLV2-14/IGLV2-23/IGKC/IGHV4-39/IGHV3-33/CLU/IGKV1-16/IGLV2-11/IGHG1/IGKV3-15/IGHV2-70/IGHV3-53 |
|  | GO:2000257 | regulation of protein activation cascade | 34 | 1.34938893782483E-26 | IGLV3-25/IGHV4-59/IGHG4/C5AR1/IGLV3-21/IGLV1-47/IGHV4-34/IGLV1-40/IGKV4-1/IGLC3/IGHG3/IGLV1-44/IGKV1-39/IGLV1-51/IGLC2/IGKV1-5/CFB/IGHV3-30/IGLV3-19/IGHV3-23/IGHG2/IGLV3-1/IGLV2-14/IGLV2-23/IGKC/IGHV4-39/IGHV3-33/CLU/IGKV1-16/IGLV2-11/IGHG1/IGKV3-15/IGHV2-70/IGHV3-53 |
|  | GO:0002673 | regulation of acute inflammatory response | 37 | 6.63638524771737E-25 | IGLV3-25/IGHV4-59/IGHG4/C5AR1/IGLV3-21/IGLV1-47/IGHV4-34/IGLV1-40/IGKV4-1/IGLC3/IGHG3/IGLV1-44/IGKV1-39/IGLV1-51/IGLC2/IGKV1-5/CFB/PTGER3/IGHV3-30/IGLV3-19/IGHV3-23/IGHG2/IGLV3-1/PIK3CG/PTGS2/IGLV2-14/IGLV2-23/IGKC/IGHV4-39/IGHV3-33/CLU/IGKV1-16/IGLV2-11/IGHG1/IGKV3-15/IGHV2-70/IGHV3-53 |
