# Supplementary material for: An integrative bioinformatics analysis for identifying hub genes associated with infection of lung samples in patients infected with SARS-CoV-2
Source: Eur J Med Res. 2021 Dec 17;26:146. doi: 10.1186/s40001-021-00609-4 (PMC8677925; doi:10.1186/s40001-021-00609-4)
Supplement: Supplementary file 5 — Additional file 5: Table S4. Ten most significantly enriched KEGG pathways in the system matrix file GSE147507 and GSE150316. [file 40001_2021_609_MOESM5_ESM.docx]

**Supplementary Table S4. Ten most significantly enriched KEGG pathways in the system matrix file GSE147507 and GSE150316.**

| The data set | ID | Descripcion | Count | P-value | P.adjust | GeneID |
| --- | --- | --- | --- | --- | --- | --- |
| GSE147507 | hsa04380 | Osteoclast differentiation | 29 | 7.23E-10 | 2.16E-07 | LILRA6/FOS/TYROBP/TNF/LCP2/LILRA5/SPI1/SIRPB1/FCGR3B/FOSB/MAP2K6/JUN/JUND/LILRB2/LILRB3/STAT1/FCGR2A/ITGB3/IRF9/IL1A/NCF4/PPP3R1/NFATC2/IL1B/TGFBR1/STAT2/MAP2K7/TGFBR2/TRAF2 |
|  | hsa04062 | Chemokine signaling pathway | 28 | 1.49E-05 | 0.00112937 | CCL8/CCL4/CCL11/WAS/CXCL10/CCL19/ARRB2/PTK2B/RAC2/FGR/CCL3/GNG5/GNG2/CCR1/CCL18/CXCL16/STAT1/RAF1/CCL2/LYN/HCK/ADCY6/ADCY4/JAK3/CXCL11/PRKCZ/BCAR1/STAT2 |
|  | hsa05135 | Yersinia infection | 22 | 1.51E-05 | 0.00112937 | FOS/WAS/TNF/LCP2/PTK2B/RAC2/MAP2K6/JUN/ACTB/FCGR2A/CCL2/ARHGEF7/NFATC2/IL1B/BAIAP2/WIPF1/CD8A/BCAR1/RPS6KA2/CD4/MAP2K7/TRAF2 |
|  | hsa04621 | NOD-like receptor signaling pathway | 27 | 1.86E-05 | 0.00112937 | OAS1/OAS3/OAS2/CARD16/CARD17/GBP5/TNF/JUN/GBP3/ITPR3/CASP4/GBP4/GBP1/STAT1/CYBB/IRF9/CCL2/ITPR1/RNF31/NOD1/IL1B/TRAF5/AIM2/IRF7/NLRP1/STAT2/TRAF2 |
|  | hsa04625 | C-type lectin receptor signaling pathway | 19 | 1.89E-05 | 0.00112937 | CLEC4E/FCER1G/CLEC4D/TNF/JUN/ITPR3/CLEC7A/BCL3/STAT1/RAF1/IRF9/ITPR1/MAPKAPK2/NFATC4/PPP3R1/NFATC2/IL1B/EGR2/STAT2 |
|  | hsa05164 | Influenza A | 25 | 5.33E-05 | 0.002657976 | OAS1/EIF2AK2/MX2/OAS3/OAS2/TPSB2/MX1/TNFSF10/CXCL10/TNF/DDX58/RSAD2/ACTB/STAT1/RAF1/SLC25A4/HLA-DRB5/IRF9/IL1A/CCL2/IFIH1/IL1B/IRF7/FDPS/STAT2 |
|  | hsa04657 | IL-17 signaling pathway | 16 | 0.000203155 | 0.008677617 | CCL11/FOS/S100A8/CXCL10/TNF/S100A9/FOSB/MMP1/JUN/JUND/LCN2/IL17C/CCL2/IL1B/TRAF5/TRAF2 |
|  | hsa05160 | Hepatitis C | 22 | 0.000252282 | 0.009429045 | IFIT1/OAS1/EIF2AK2/MX2/OAS3/OAS2/MX1/CXCL10/TNF/DDX58/RSAD2/CLDN5/CLDN2/STAT1/RAF1/IRF9/CCND1/PSME3/IRF7/PPP2R2C/STAT2/TRAF2 |
|  | hsa05169 | Epstein-Barr virus infection | 26 | 0.000319844 | 0.009917865 | OAS1/EIF2AK2/OAS3/ISG15/OAS2/CXCL10/TNF/MAP2K6/DDX58/JUN/STAT1/HLA-DRB5/IRF9/LYN/PSMC5/GADD45G/JAK3/CCND1/TRAF5/IRF7/DDB2/PSMD2/HLA-A/STAT2/MAP2K7/TRAF2 |
|  | hsa05163 | Human cytomegalovirus infection | 29 | 0.000335377 | 0.009917865 | CCL4/TNF/PTK2B/RAC2/MAP2K6/CCL3/GNG5/GNG2/ITPR3/CCR1/RAF1/ITGB3/CCL2/ITPR1/ADCY6/ADCY4/NFATC4/PPP3R1/CCND1/NFATC2/IL1B/ATF6B/TRAF5/RPS6KB2/TSC1/BCAR1/HLA-A/TRAF2 |
| GSE150316 | **/** | **/** | **/** | **/** | **/** | **/** |
